# Changes in Mortality During the COVID-19 Pandemic in Japan: Descriptive Analysis of National Health Statistics up to 2022

**DOI:** 10.2188/jea.JE20240158

**Published:** 2025-03-05

**Authors:** Hirokazu Tanaka, Shuhei Nomura, Kota Katanoda

**Affiliations:** 1 Division of Population Data Science, National Cancer Center Institute for Cancer Control, Tokyo, Japan; 2 Keio University Global Research Institute (KGRI), Tokyo, Japan; 3 Department of Health Policy and Management, School of Medicine, Keio University, Tokyo, Japan; 4 Division of Prevention, National Cancer Center Institute for Cancer Control, Tokyo, Japan

**Keywords:** age-standardized mortality rate, COVID-19, senility, excess death, Vital Statistics

## Abstract

**Background:**

Amidst the global coronavirus disease 2019 (COVID-19) pandemic, Japan has faced a significant public health challenge, evident from the significant increase in mortality rates since 2021. This study described the variations in all-cause and cause-specific changes in mortality up to 2022 in Japan.

**Methods:**

This study used official Vital Statistics from the Ministry of Health, Labour and Welfare (MHLW) to assess the impact of the pandemic on mortality trends. An analysis of all-cause and cause-specific age-standardized mortality rates (ASMRs) from 1995 to 2022 was conducted, employing the 2015 Japan Standard Population. Sex- and cause-specific ASMRs for a particular year were compared with those from the preceding year to assess annual changes.

**Results:**

Among men, the annual all-cause ASMR per 100,000 people increased from 1,356.3 in 2021 to 1,437.8 in 2022 (6.0% increase). Among women, the annual all-cause ASMR increased from 722.1 in 2021 to 785.8 in 2022 (6.5% increase). Compared with the period 2020 to 2021, COVID-19 (+29.1 per 100,000 people for men and +13.4 per 100,000 people for women), senility (+14.1 per 100,000 people for men and +12.5 per 100,000 people for women), heart disease, malignant neoplasms (for women) and “other causes not classified as major causes” substantially contributed to the increase in all-cause ASMR from 2021 to 2022.

**Conclusion:**

Further long-term monitoring from 2023 onwards is necessary, especially for conditions like senility, cardiovascular disease, and cancer, which may have long-term effects due to changes in healthcare settings, even though the strong countermeasures against COVID-19 were lifted in 2023.

## INTRODUCTION

In Japan, life expectancy has increased in 2020 (+0.22 years for men and +0.30 years for women compared to 2019).^1^ This sharply contrasts with many other countries, where coronavirus disease 2019 (COVID-19) led to decreases in life expectancy as early as 2020.^2^^,^^3^ However, in 2021, a notable shift in mortality trends occurred in Japan, with all-cause age-standardized mortality rates (ASMR) increasing by 2% compared to 2020.^4^ This change highlights the contrasting patterns observed in Japan; namely, the low impact of COVID-19 in 2020 led to increased life expectancy, followed by a delayed increase in mortality in 2021. This pattern differs from the immediate increase in mortality seen in other nations since 2020.^2^^,^^3^

Following the onset of the pandemic, Japan, along with the rest of the world, initiated national vaccination program as early as in 2021, after the highest surge in 2022, it gradually lifted entry restrictions, and eased other border and movement restrictions.^5^ In May 2023, the World Health Organization (WHO) announced the end of the emergency declaration for COVID-19. Despite this milestone, a thorough assessment of the mortality impact attributable to COVID-19 and other diseases during this period remains necessary, since the full scope of indirect health impacts, such as delayed medical treatments and psychological effects, has not yet been fully understood.^6^ Such an analysis is essential for evaluating the resilience of healthcare systems to pandemics and the extent of damage, to identify societal vulnerabilities. Our prior analyses revealed a reversal in the declining trend of all-cause ASMR in 2021,^4^ the first occurrence of such an event since the Great East Japan Earthquake and the subsequent tsunami in 2011, which resulted in significant fatalities. The increase in ASMR in 2021 was likely attributable to factors such as COVID-19, senility, and cardiovascular diseases.^4^ Furthermore, because Japan experienced the largest number of COVID-19 cases in 2022,^7^ this is likely to have further implications for mortality trends afterward. This study aimed to comprehensively explore the changes in all-cause ASMR and their cause-specific contributions up to 2022, to provide updated insights.

## METHODS

First, we assessed the daily trends in COVID-19 cases and death counts in Japan by sourcing open data by the Ministry of Health, Labour and Welfare (MHLW).^7^ Second, we examined the changes in the annual all-cause ASMR for the period 1995–2022 as the main analysis. ASMR calculations used the 2015 Japan Standard Population for standardization.^8^ We also investigated the changes in all-cause crude mortality by 5-year groups and the annual ASMR by 47 prefectures between 2017 and 2022 to assess differences in age groups and regions. For calculating ASMR by prefectures, we used the variant method of direct standardization (rounding to ≥85 years) because the 5-year population data by prefecture are not available for those aged 85–89 years and 90–94 years.^8^ We calculated 95% confidence intervals for each value. However, we did not present these due to their extremely narrow range, which is a result of our analysis being based on comprehensive national mortality data for the entire Japanese population rather than a sample. This analysis utilized open data on death counts, segmented into 5-year groups, as documented in the Vital Statistics registry maintained by the MHLW.^1^ Additionally, relevant population data were obtained from open datasets, specifically the Population Census and Vital Statistics records. We further quantified the annual percent changes in ASMRs to delineate the variations preceding and during the COVID-19 pandemic (2020–2022). Unless specified otherwise, the ASMR is per 100,000 population for all causes.

Third, our analysis included cause-specific ASMR as well, adhering to the International Classification of Diseases, 10th revision (ICD-10) categories: infectious and parasitic diseases (A00–B99), malignant neoplasms (C00–C96), heart diseases (I01–I02.0, I05–I09, I20–I25, I27, I30–I52), cerebrovascular diseases (I60–I69), pneumonia (J12–J18), liver disease (K70–K76), senility (R54), accidents (V01–X59), suicide (X60–X84), and COVID-19 (U07), with an additional focus on major site-specific cancers (eg, stomach cancer: C16). These major categories were selected based on leading causes of death in the official mortality statistics from the MHLW.^1^ To evaluate the contribution of the specific causes to annual all-cause ASMR fluctuations, we compared the yearly cause-specific ASMR changes for twelve consecutive periods from 2010–2011 to 2021–2022. The ASMR and cause-specific ASMR changes were calculated as described previously.^4^ Briefly, differences in changes in cause-specific ASMR between 2021 and 2022 were calculated as (ASMR_2022_ − ASMR_2021_) for each cause. These differences were then represented as stacked bar charts for each period.^4^

## RESULTS

The daily trends in confirmed COVID-19 cases and COVID-19 deaths in Japan from January 2020 to date are presented in eFigure 1. The annual number of COVID-19 cases was recorded as 234,109 in 2020, escalating to 1,492,874 in 2021 and further surging to 27,219,936 in 2022, with a peak observed in August and September 2022.

**Figure 1.**
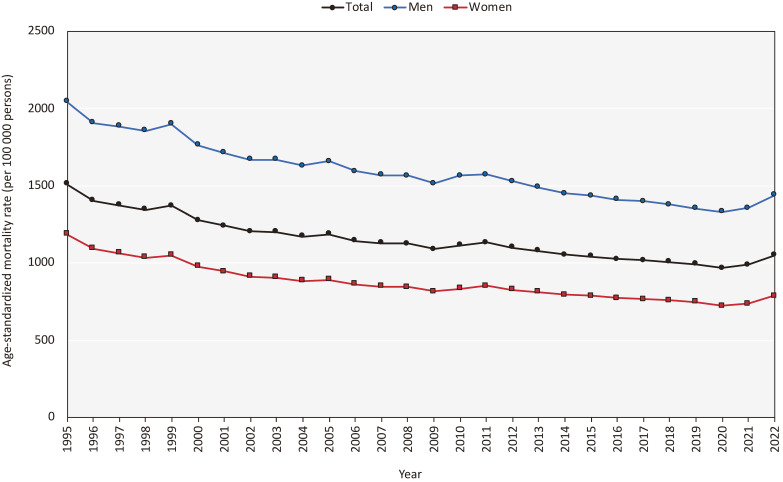
Trends in all-cause age-standardized mortality rates in Japan

The annual all-cause ASMRs generally declined from 1995 through 2020, followed by an increase in 2021 for both sexes (Figure 1). Specifically, in men, the all-cause ASMR per 100,000 population increased from 1,328.8 in 2020 to 1,356.3 in 2021 (2.1% increase) and further to 1,437.8 in 2022 (6.0% increase). In women, the annual all-cause ASMR increased from 722.1 in 2020 to 737.9 in 2021 (2.2% increase) and to 785.8 in 2022 (6.5% increase) (eTable 1 for ASMR values and 2 for their percent changes). Regarding the changes in mortality by age, almost all age groups showed an increase in crude mortality between 2021 and 2022 (eTable 3). Moreover, the increase was prominent among those aged ≥70 years (7–11% increase). Truncated age-standardized mortality analysis (ASMR stratified by 0–39 years, 40–69 years, and ≥70 years) showed large mortality increases between 2021 and 2022 in heart diseases among those aged ≥70 years (+6.0% for both sexes) and suicide among those aged 40–69 years (+8.9% for both sexes) (eTable 4). In the analysis for ASMR by prefectures, all-cause ASMR increased in all prefectures (minimum: +2.9% in Niigata and maximum: +9.3% in Kumamoto for both sexes) between 2021 and 2022, with most of the increases by prefectures between 2021 and 2022 exceeding those between 2020 and 2021 (eTable 5). Large mortality increases were not observed in urban regions such as Tokyo (6.5% for both sexes) and Osaka (5.9%).

**Figure 2.**
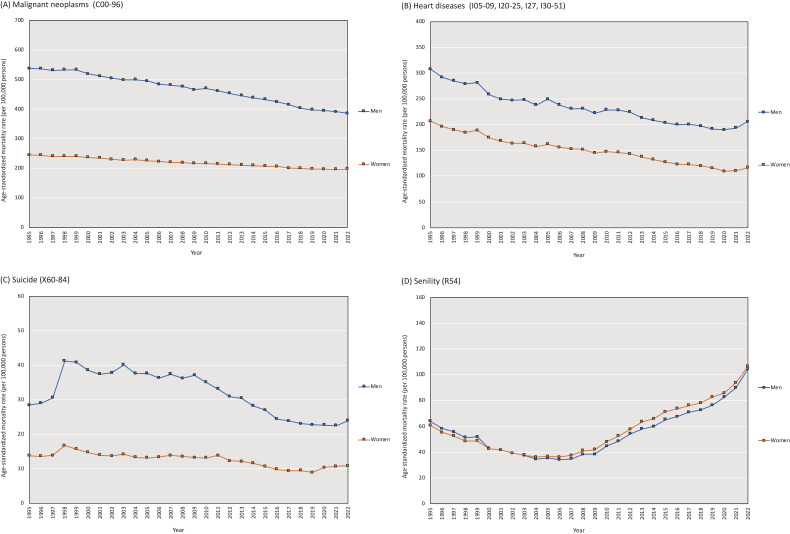
Trends in selected cause-specific age-standardized mortality rates in Japan for (**A**) malignant neoplasms, (**B**) heart diseases, (**C**) suicide, and (**D**) senility

Figure 2 presents the trends in annual ASMR for malignant neoplasms, heart disease, suicide, and senility. In 2022, the annual ASMR for malignant neoplasms decreased from 2021 by 1.4% in men but increased by 1.0% in women. The trends in cause-specific ASMRs (eTable 1) and annual percent changes (eTable 2) highlight the divergent trends in ASMR changes by sex for malignant neoplasms. The trends in annual ASMRs for malignant neoplasms according to the cancer site from 1995 to 2022 are presented in eFigure 2. For women, notable increases in annual ASMRs were observed for breast cancer (6.1% increase between 2021 and 2022), colorectal cancer (1.2% increase), uterine cancer (4.2% increase), and pancreatic cancer (1.7% increase), underscoring significant relative and absolute growth in the annual ASMRs in these areas.

**Figure 3.**
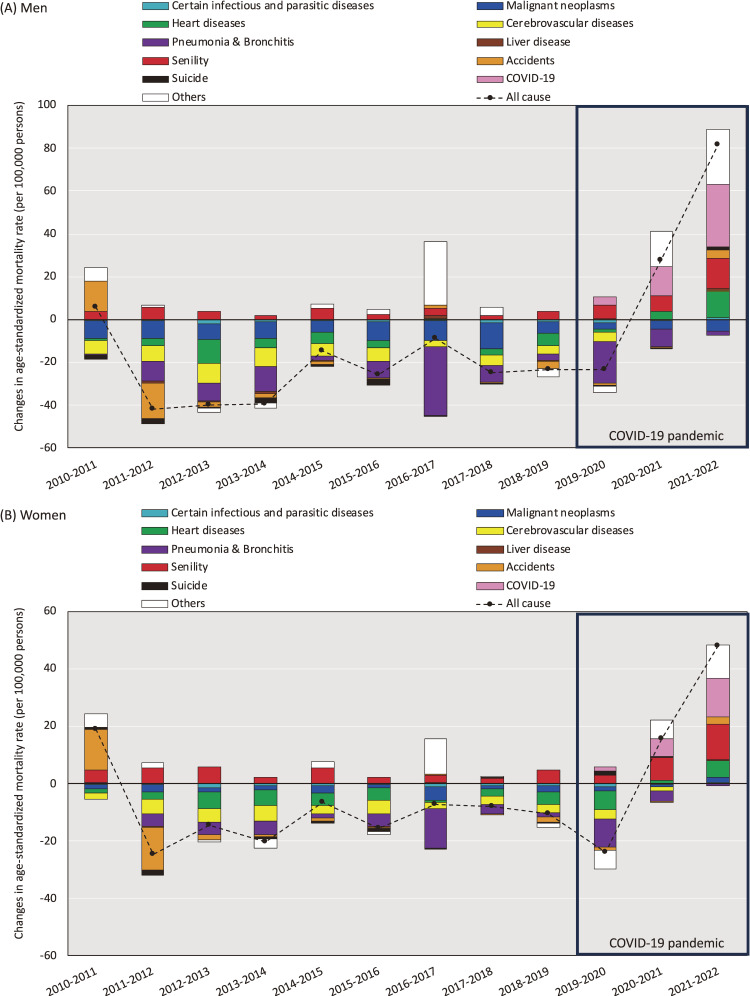
Cause-specific contribution to changes in all-cause age-standardized mortality rates (annual comparisons with previous year) for (**A**) men and (**B**) women. Differences in changes in ASMR between 2021 and 2022 were calculated as (ASMR_2022_ − ASMR_2021_) for each cause-specific death, where ASMR = age standardized mortality rate per 100,000 persons.

The primary factors exacerbating annual ASMRs from 2021 to 2022 included COVID-19, with an absolute annual ASMR increment of 29.1 per 100,000 people for men and 13.4 per 100,000 people for women, and senility, with an increment of 14.1 per 100,000 people for men and 12.5 per 100,000 people for women in comparison to the previous year (Figure 3). Furthermore, heart disease significantly contributed to an increase in annual ASMRs between 2021 and 2022. Heart disease exhibited an absolute annual ASMR increment of 11.8 per 100,000 people for men and 5.7 per 100,000 people for women, compared with that in the previous year.

Compared with the change observed between 2020 and 2021, a more substantial contribution to the increase in annual all-cause ASMR from COVID-19, senility, heart disease, and “other causes not classified as major causes” was noted from 2021 to 2022. In men, we identified that suicide, with an increment of 1.5 per 100,000 people, was the cause of death that exacerbated annual ASMRs from 2021 to 2022, whereas a decrease of 0.2 per 100,000 people was observed from 2020 to 2021. In women, we identified that malignant neoplasms, with an absolute annual ASMR increment of 1.9 per 100,000 people, were the cause of death that exacerbated annual ASMRs from 2021 to 2022, whereas a decrease of 0.8 per 100,000 people (from 2020 to 2021) alleviated annual ASMRs from 2010 to 2021 (decreases of approximately 1.0–5.0 per 100,000 people).

## DISCUSSION

Japan witnessed the highest annual number of COVID-19 cases and deaths, and all-cause ASMR in 2022 since the onset of the COVID-19 pandemic in 2020. Notably, the increase in annual ASMR, estimated at approximately 6% from 2021 to 2022, marked the first significant rise in the study period. Taking long-term trends in annual ASMR in Japan into consideration,^8^ about a 6% increase in annual all-cause ASMR was observed for the first time in a half-century. Moreover, all-cause crude mortality increased in almost all age groups, and all prefectures showed an increase in ASMR between 2021 and 2022. We found the variation in mortality increasing across prefectures; however, it is difficult to discuss the detailed mechanism of which factor was attributed to the variation using our descriptive data. Further analysis is necessary for clarifying prefecture differences in mortality by the COVID-19 measure adherence, such as vaccinations, mask-wearing, and social distancing, including the baseline performance of healthcare systems and social determinants of health.

We identified a pronounced increase in annual ASMRs due to COVID-19, senility, and heart disease, which contributed to an increase in annual all-cause ASMR in 2022. This trend was consistent with reports from 2021.^4^ In addition to the direct impact of widespread COVID-19 transmission, the increase in ASMR caused by senility might be influenced by a range of possible factors, and the causes of this increase are still under debate. Potential contributing factors could include the effects of physical and psychological declines resulting from containment measures against COVID-19^9^^–^^11^ and possibly failures in coordinating hospital admissions from homes or care facilities, potentially leading to a lack of medical care before death and possibly resulting in inaccurate determination of the cause of death. This situation may have led to an increase in “senility” as a cause of death, potentially involving various unspecified conditions (sometimes referred to as “garbage codes” in epidemiological literature).^12^ Throughout the pandemic, the number of deaths associated with senescence at home has increased since 2020.^13^^–^^15^ While it is challenging to conclusively determine the specific reasons for the increase in senility-related deaths, the notable increase after 2020 warrants attention and further investigation.

Increasing mortality owing to heart disease emerged as an important public health issue during the pandemic in Japan. Research has indicated a correlation between COVID-19 and the onset of acute myocardial infarction and ischemic stroke,^16^^,^^17^ which could have directly contributed to an increase in cardiovascular disease-related deaths since 2021.^15^ Furthermore, the pandemic has indirectly affected patient care by causing delays in emergency transport and hospital admissions of patients requiring urgent medical attention. Specifically, prolonged door-to-balloon times have been reported for primary percutaneous coronary intervention,^18^^,^^19^ and delays in the presentation and treatment of ST-elevation myocardial infarction^20^ during the pandemic in certain clinical settings. Although these delays have not demonstrably deteriorated the treatment outcomes at individual hospitals, the cumulative effect of these healthcare disruptions may have contributed to the national increase in cardiovascular mortality.

Importantly, in 2022, the annual ASMR for malignant neoplasms among women in Japan increased by 1.0% from 2021. This observation was significant considering the consistent long-term decline in ASMRs observed over half a century, with a few exceptions, such as in 2004. The increase in ASMRs for breast, colorectal, and uterine cancers imply changes in healthcare provisions, particularly cancer screening and treatment protocols, which have been in effect since 2020. However, these findings warrant cautious interpretation. Participation in cancer screening declined significantly by approximately 20% in 2020 for individuals aged ≥40 years.^21^ Although there was a slight recovery in 2021 and 2022, the decreased rate of early detection, as represented by reduced cancer screenings, may correlate with the mortality trends observed in 2022, especially concerning cancers prevalent among women and colorectal cancer.^22^ The decline in the proportion of early-stage cancer diagnoses in 2020 further supports this hypothesis.^23^

For men, we revealed an increase in ASMR from suicide in 2022, marking the first rise since 2009, which followed the collapse of Lehman Brothers. The escalation in suicide rates post-2020 represents an important social issue in Japan, with a pronounced impact on women.^4^ Their study demonstrated a significant surge in ASMR owing to suicide among women, with an increase of 16.6% from 2019 to 2020^4^; this upward trend persisted throughout 2022 without any observed decline. This pattern aligns with the estimates of monthly excess deaths until January 2023.^24^^,^^25^

Discussions of excess death were essential in interpreting changes in mortality during the global COVID-19 pandemic. Here, our discussion also involves evaluating excess mortality through the “Excess and Exiguous Deaths Dashboard in Japan,” managed by the National Institute of Infectious Diseases, Japan.^26^ This platform has been instrumental in publishing a series of studies on excess deaths for the Japanese population,^13^^–^^15^^,^^25^^,^^27^^,^^28^ providing open access to their estimations through the dashboard.^26^ According to the estimations,^26^ 3.3–7.6% of the total number of all-cause deaths in 2022 (*n* = 1,569,050; reported in the Vital Statistics) could be attributed to estimated excess deaths (estimates ranged from 51,056 to 119,401 in 2022; eTable 6) associated with the COVID-19 pandemic. This highlights the critical importance of continuous surveillance and timely updates to existing assessments as more recent data becomes available, to understand the long-term effects of the COVID-19 pandemic in Japan and guide the development of strategies for the normalization of healthcare services post-pandemic.

This study had some limitations, particularly its concept as a descriptive investigation of national mortality statistics. First, the update of the principal cause of death criteria by the MHLW in January 2017 caused to rapid changes in some cause-specific ASMR.^29^ For example, we observed a significant shift in the cause of mortality from “pneumonia & bronchitis” (a decline) to “others” (an increase) during 2016–2017 for both sexes. However, these updates in the primary cause of death did not significantly affect the assessment of the COVID-19 pandemic in this study. Second, our analysis did not include a causal analysis between related factors and changes in ASMR because this is a descriptive analysis. Further study is necessary to address the impact of the COVID-19 pandemic on mortality using the combination of clinical data and health surveys such as the national cancer registry for the Japanese populations.

### Conclusions

In Japan, the number of COVID-19 cases/deaths, all-cause ASMR, and excess deaths in 2022 were the largest in the 3 years since the COVID-19 pandemic began. Further long-term monitoring from 2023 onwards is necessary, especially for conditions like senility, cardiovascular disease, and cancer, which may have long-term effects due to changes in healthcare settings, even though the COVID-19 measures were lifted in 2023.

## SUPPLEMENTARY MATERIAL

eTable 1. Trends in all-cause and cause-specific age-standardized mortality rate calculated using the 2015 Japanese Standard population, 1995–2022 (per 100,000 persons)

eTable 2. The percent changes in all-cause and cause-specific age-standardized mortality rate in comparison with the previous yaer, 1995–2022 (%)^*^

eTable 3. Changes in crude mortality by 5-year groups (per 100,000 persons)

eTable 4. Changes in cause-specific truncated age-standardized mortality (per 100,000 persons)

eTable 5. Age-standardized mortality rate by prefectures calculated using the 2015 Japanese Standard population (per 100,000 persons, rounding 85 years and over)

eTable 6. Number of observed deaths, excess deaths, and exiguous deaths^*^ in Japan between 2017 and 2022 (for both sexes)

eFigure 1. Trends in the daily number of (**A**) reported COVID-19 cases and (**B**) COVID-19 deaths in Japan between 2020 and 2022

eFigure 2. Trends in cancer age-standardized mortality rates by cancer site between 1995 and 2022
