# Supplementary material for: Changes in Mortality During the COVID-19 Pandemic in Japan: Descriptive Analysis of National Health Statistics up to 2022
Source: J Epidemiol. 2025 Mar 5;35(3):154–9. doi: 10.2188/jea.JE20240158 (PMC11821379; doi:10.2188/jea.JE20240158)
Supplement: Supplementary file 1 [file je-35-154-s001.pdf]

eTable 1. Trends in all-cause and cause-specific age-standardized mortality rate calculated using the 2015 Japanese Standard population, 1995–2022 (per 100,000 persons)

Simple cause of death classification		-	01000	02100	09200	09300	10200	11300	18100	20100	20200	
Sex	Year	All-cause	Infectious and parasitic diseases	Malignant neoplasms	Heart diseases	Cerebrovascular diseases	Pneumonia & Bronchitis	Liver disease	Senility	Accidents	Suicide	COVID-19
Both	1995	1510.7	28.3	359.1	245.7	266.5	163.5	20.7	61.8	57.2	20.4	-
Both	1996	1402.2	25.5	358.6	233.3	242.8	138.2	19.3	56.2	49.0	20.7	-
Both	1997	1373.6	25.2	353.6	226.7	229.5	146.1	18.9	53.4	47.4	21.5	-
Both	1998	1345.8	24.3	354.3	221.4	217.0	140.6	17.9	49.3	46.8	28.1	-
Both	1999	1370.3	23.2	354.1	225.1	210.1	157.7	18.0	49.5	47.3	27.3	-
Both	2000	1277.9	25.1	347.6	208.3	190.1	137.9	16.9	42.7	45.1	25.9	-
Both	2001	1239.5	26.5	343.4	200.9	180.9	128.8	16.3	41.7	44.0	24.9	-
Both	2002	1204.3	25.0	338.1	197.4	170.3	124.8	15.5	39.3	41.9	25.1	-
Both	2003	1200.4	23.8	334.3	198.4	165.4	129.1	15.5	37.6	41.2	26.4	-
Both	2004	1172.4	23.9	336.1	190.6	155.1	123.6	15.4	35.9	39.6	24.7	-
Both	2005	1186.6	25.1	331.1	197.3	152.0	131.2	15.6	36.4	40.2	24.7	-
Both	2006	1144.9	24.9	325.8	189.6	140.6	124.8	15.2	35.7	37.6	24.2	-
Both	2007	1128.3	24.5	323.6	184.9	133.5	121.8	14.8	36.9	36.6	24.9	-
Both	2008	1126.0	24.3	321.4	184.7	128.6	121.8	14.6	40.7	35.9	24.3	-
Both	2009	1089.8	23.7	314.9	177.1	119.3	113.4	14.1	41.2	34.7	24.6	-
Both	2010	1115.9	23.9	316.0	180.8	117.2	116.8	14.0	47.5	36.7	23.5	-
Both	2011	1131.2	23.6	312.4	179.6	113.6	117.4	14.0	51.8	50.8	23.0	-
Both	2012	1101.1	23.3	308.0	176.7	107.6	111.8	13.4	57.2	35.2	21.1	-
Both	2013	1079.1	21.7	304.4	169.1	101.4	106.6	13.2	62.6	33.1	20.8	-
Both	2014	1052.6	21.0	300.7	163.9	94.7	100.0	12.8	64.7	32.0	19.5	-
Both	2015	1043.7	20.3	297.3	159.2	90.7	98.4	12.6	70.3	30.8	18.4	-
Both	2016	1025.8	19.6	293.7	155.2	85.6	93.1	12.6	72.7	30.1	16.7	-
Both	2017	1018.7	18.8	287.4	155.0	83.2	72.6	13.3	75.4	30.8	16.3	-
Both	2018	1005.5	17.8	281.9	152.5	79.5	68.3	13.4	77.3	30.8	16.0	-
Both	2019	992.6	16.9	278.9	147.8	76.3	66.7	13.3	81.9	28.6	15.5	-
Both	2020	968.4	15.6	276.8	143.2	72.4	53.5	13.5	85.8	27.3	16.2	2.5
Both	2021	989.6	15.3	275.0	145.2	71.6	48.4	13.7	93.8	26.8	16.3	11.8
Both	2022	1051.0	16.0	273.9	153.5	72.0	47.5	14.2	106.8	29.7	17.1	30.7
Male	1995	2044.3	38.6	536.9	308.0	326.9	246.8	30.0	64.0	81.3	28.4	-
Male	1996	1906.4	35.0	536.5	291.6	298.1	215.2	28.1	58.3	72.9	29.0	-
Male	1997	1884.5	34.6	530.6	284.8	284.5	228.2	27.7	55.6	70.7	30.6	-
Male	1998	1856.6	34.9	532.5	279.0	270.9	219.2	26.7	51.2	69.9	41.2	-
Male	1999	1900.6	36.7	532.5	281.3	264.3	247.7	26.9	51.8	71.2	40.8	-
Male	2000	1760.8	34.2	519.2	258.3	236.1	217.2	24.9	42.8	67.3	38.5	-
Male	2001	1710.5	33.2	511.0	249.3	226.8	204.7	23.9	41.6	65.6	37.4	-
Male	2002	1669.8	31.3	504.5	247.3	213.7	199.8	22.7	39.2	62.4	37.8	-
Male	2003	1668.5	31.9	498.5	248.5	209.1	206.1	22.7	37.4	60.9	40.1	-
Male	2004	1628.9	32.0	499.9	238.2	196.0	200.0	22.6	34.6	58.6	37.6	-
Male	2005	1659.8	32.9	494.4	249.2	194.3	213.4	22.6	35.3	60.0	37.6	-
Male	2006	1594.2	32.3	483.8	238.4	179.5	200.7	22.2	34.1	56.0	36.3	-
Male	2007	1568.9	31.6	480.6	230.8	170.6	197.3	21.5	34.7	53.3	37.4	-
Male	2008	1565.1	31.5	476.0	231.6	164.5	196.6	21.0	38.4	52.5	36.2	-
Male	2009	1515.6	30.6	465.0	222.3	153.7	183.2	20.4	38.3	51.4	37.1	-
Male	2010	1564.6	31.0	469.4	228.9	153.7	191.9	20.3	44.6	54.3	35.0	-
Male	2011	1570.5	30.3	461.5	227.9	147.3	191.3	20.4	48.4	68.2	33.1	-
Male	2012	1528.7	29.7	453.3	224.6	139.7	182.1	19.6	54.2	51.6	30.9	-
Male	2013	1489.0	27.7	446.0	213.4	130.4	173.9	19.3	58.0	48.8	30.4	-
Male	2014	1449.7	26.7	438.4	208.7	121.7	162.3	18.4	60.0	46.7	28.2	-
Male	2015	1434.9	26.0	433.0	203.6	116.0	160.3	18.1	65.2	45.1	27.0	-
Male	2016	1409.2	25.0	424.4	199.9	110.0	152.6	18.2	67.4	44.1	24.3	-
Male	2017	1400.3	24.4	415.1	200.3	107.4	120.3	19.6	70.9	45.5	23.8	-
Male	2018	1375.6	23.0	402.9	197.4	102.3	112.5	19.7	72.7	45.3	23.0	-
Male	2019	1352.3	22.1	397.7	191.4	98.1	109.6	19.6	76.4	41.9	22.7	-
Male	2020	1328.8	20.4	394.7	190.1	93.8	90.1	20.2	82.7	40.8	22.6	3.8
Male	2021	1356.3	19.8	390.8	193.9	93.7	81.9	20.2	90.0	40.2	22.5	17.5
Male	2022	1437.8	20.9	385.5	205.7	94.4	80.1	21.2	104.2	43.9	23.9	46.7
Female	1995	1184.7	21.6	244.4	206.7	230.4	121.8	13.1	60.8	40.6	13.7	-
Female	1996	1092.6	19.3	243.6	196.3	209.6	100.3	12.2	55.3	32.6	13.6	-
Female	1997	1063.0	19.1	240.0	189.9	196.6	106.3	11.7	52.6	31.4	13.8	-
Female	1998	1033.8	19.2	240.0	184.7	184.8	102.4	10.8	48.5	31.1	16.7	-
Female	1999	1049.9	20.2	240.4	189.1	177.4	114.7	10.8	48.7	31.4	15.6	-
Female	2000	976.3	19.3	236.1	174.7	161.3	99.1	10.2	42.5	29.9	14.7	-
Female	2001	945.2	18.9	234.6	167.9	152.1	92.1	9.7	41.6	29.2	13.9	-
Female	2002	913.0	18.2	230.0	163.5	142.9	88.4	9.4	39.1	27.9	13.6	-
Female	2003	907.0	18.6	227.4	164.0	137.8	91.8	9.3	37.6	27.7	14.1	-
Female	2004	884.7	18.8	229.0	157.6	129.2	86.7	9.3	36.2	26.4	13.3	-
Female	2005	891.6	20.1	225.0	161.8	125.3	91.8	9.4	36.6	26.7	13.1	-
Female	2006	862.4	20.2	222.3	156.1	115.7	88.1	9.1	36.1	25.3	13.3	-
Female	2007	848.0	19.9	220.0	152.7	109.1	85.1	9.0	37.4	25.1	13.8	-
Female	2008	845.1	19.8	219.0	151.5	104.6	84.9	9.1	41.1	24.4	13.5	-
Female	2009	814.3	19.4	215.5	144.6	96.2	78.3	8.6	41.9	23.3	13.2	-
Female	2010	833.7	19.5	216.2	147.4	93.3	80.2	8.5	48.1	25.0	13.1	-
Female	2011	852.6	19.4	214.5	145.9	91.1	80.8	8.6	52.4	39.2	13.8	-
Female	2012	828.0	19.3	211.9	143.2	86.0	76.5	8.0	57.7	24.4	12.2	-
Female	2013	813.6	18.0	210.3	137.3	81.2	72.5	7.9	63.6	22.6	12.1	-
Female	2014	793.4	17.5	208.8	131.8	75.6	67.8	7.9	65.8	21.9	11.5	-
Female	2015	787.2	16.7	206.6	127.4	72.6	66.4	7.8	71.4	21.1	10.6	-
Female	2016	771.7	16.2	205.6	123.1	68.0	61.8	7.7	73.7	20.5	9.8	-
Female	2017	764.4	15.3	200.7	122.6	65.7	47.8	8.0	76.2	21.0	9.4	-
Female	2018	756.5	14.5	199.6	120.1	62.8	44.7	7.9	78.2	20.9	9.5	-
Female	2019	746.0	13.6	197.6	115.7	59.9	43.3	7.8	82.8	19.4	8.9	-
Female	2020	722.1	12.4	196.4	109.2	56.4	33.4	7.7	85.8	18.2	10.3	1.5

eTable 2. The percent changes in all-cause and cause-specific age-standardized mortality rate in comparison with the previous yaer, 1995–2022 (%)*

Simple cause of death classification		-	01000	02100	09200	09300	10200	11300	18100	20100	20200	
Sex	Year	All-cause	Infectious and parasitic diseases	Malignant neoplasms	Heart diseases	Cerebrovascular diseases	Pneumonia & Bronchitis	Liver disease	Senility	Accidents	Suicide	COVID-19
Both	1995	-	-	-	-	-	-	-	-	-	-	-
Both	1996	-7.2%	-9.8%	-0.1%	-5.0%	-8.9%	-15.5%	-6.4%	-9.0%	-14.2%	1.2%	-
Both	1997	-2.0%	-1.1%	-1.4%	-2.8%	-5.5%	5.8%	-2.3%	-5.0%	-3.3%	4.1%	-
Both	1998	-2.0%	-3.5%	0.2%	-2.3%	-5.4%	-3.8%	-5.2%	-7.8%	-1.3%	30.8%	-
Both	1999	1.8%	-4.5%	-0.1%	1.7%	-3.2%	12.2%	0.5%	0.6%	1.1%	-2.9%	-
Both	2000	-6.7%	8.1%	-1.8%	-7.5%	-9.5%	-12.5%	-5.8%	-13.8%	-4.5%	-5.1%	-
Both	2001	-3.0%	5.5%	-1.2%	-3.6%	-4.8%	-6.6%	-4.1%	-2.4%	-2.6%	-3.8%	-
Both	2002	-2.8%	-5.5%	-1.5%	-1.8%	-5.8%	-3.1%	-4.5%	-5.9%	-4.6%	0.5%	-
Both	2003	-0.3%	-5.0%	-1.1%	0.5%	-2.9%	3.4%	-0.2%	-4.2%	-1.8%	5.4%	-
Both	2004	-2.3%	0.4%	0.5%	-3.9%	-6.2%	-4.2%	-0.6%	-4.5%	-3.9%	-6.4%	-
Both	2005	1.2%	5.1%	-1.5%	3.5%	-2.1%	6.1%	1.1%	1.3%	1.5%	-0.1%	-
Both	2006	-3.5%	-0.9%	-1.6%	-3.9%	-7.5%	-4.9%	-2.6%	-1.9%	-6.3%	-2.1%	-
Both	2007	-1.5%	-1.6%	-0.7%	-2.5%	-5.0%	-2.4%	-2.4%	3.4%	-2.8%	3.1%	-
Both	2008	-0.2%	-0.9%	-0.7%	-0.1%	-3.6%	0.0%	-1.3%	10.1%	-1.9%	-2.7%	-
Both	2009	-3.2%	-2.4%	-2.0%	-4.1%	-7.3%	-6.9%	-3.5%	1.4%	-3.3%	1.3%	-
Both	2010	2.4%	0.7%	0.3%	2.1%	-1.8%	3.0%	-0.5%	15.2%	5.8%	-4.3%	-
Both	2011	1.4%	-1.0%	-1.1%	-0.7%	-3.1%	0.5%	0.1%	9.0%	38.5%	-2.3%	-
Both	2012	-2.7%	-1.6%	-1.4%	-1.6%	-5.2%	-4.8%	-4.5%	10.4%	-30.7%	-8.3%	-
Both	2013	-2.0%	-6.5%	-1.2%	-4.3%	-5.8%	-4.6%	-1.7%	9.6%	-5.9%	-1.4%	-
Both	2014	-2.5%	-3.3%	-1.2%	-3.1%	-6.5%	-6.3%	-2.8%	3.3%	-3.5%	-6.3%	-
Both	2015	-0.8%	-3.4%	-1.1%	-2.8%	-4.3%	-1.6%	-1.5%	8.8%	-3.7%	-5.5%	-
Both	2016	-1.7%	-3.3%	-1.2%	-2.5%	-5.6%	-5.3%	-0.5%	3.4%	-2.3%	-9.0%	-
Both	2017	-0.7%	-4.5%	-2.1%	-0.1%	-2.8%	-22.0%	6.3%	3.8%	2.4%	-2.6%	-
Both	2018	-1.3%	-5.2%	-1.9%	-1.6%	-4.5%	-5.8%	0.3%	2.5%	-0.2%	-2.1%	-
Both	2019	-1.3%	-4.8%	-1.1%	-3.1%	-4.1%	-2.4%	-1.0%	5.9%	-7.1%	-2.7%	-
Both	2020	-2.4%	-7.8%	-0.8%	-3.1%	-5.1%	-19.7%	2.0%	4.8%	-4.3%	4.4%	-
Both	2021	2.2%	-2.1%	-0.6%	1.4%	-1.0%	-9.7%	1.2%	9.3%	-1.9%	0.5%	380.0%
Both	2022	6.2%	4.8%	-0.4%	5.8%	0.6%	-1.8%	3.9%	13.9%	10.7%	4.9%	160.7%
Male	1995	-	-	-	-	-	-	-	-	-	-	-
Male	1996	-6.7%	-9.3%	-0.1%	-5.3%	-8.8%	-12.8%	-6.3%	-8.9%	-10.4%	2.1%	-
Male	1997	-1.2%	-1.2%	-1.1%	-2.3%	-4.5%	6.1%	-1.5%	-4.6%	-3.0%	5.4%	-
Male	1998	-1.5%	0.8%	0.4%	-2.1%	-4.8%	-4.0%	-3.8%	-7.9%	-1.1%	34.9%	-
Male	1999	2.4%	5.3%	0.0%	0.8%	-2.4%	13.0%	1.0%	1.1%	1.8%	-1.1%	-
Male	2000	-7.4%	-6.7%	-2.5%	-8.2%	-10.7%	-12.3%	-7.4%	-17.3%	-5.4%	-5.5%	-
Male	2001	-2.9%	-3.2%	-1.6%	-3.5%	-3.9%	-5.8%	-4.3%	-2.7%	-2.6%	-3.0%	-
Male	2002	-2.4%	-5.6%	-1.3%	-0.8%	-5.7%	-2.4%	-4.8%	-5.8%	-4.9%	1.0%	-
Male	2003	-0.1%	1.9%	-1.2%	0.5%	-2.2%	3.1%	0.0%	-4.6%	-2.4%	6.1%	-
Male	2004	-2.4%	0.2%	0.3%	-4.1%	-6.3%	-2.9%	-0.7%	-7.7%	-3.8%	-6.2%	-
Male	2005	1.9%	3.0%	-1.1%	4.6%	-0.8%	6.7%	0.3%	2.3%	2.5%	0.1%	-
Male	2006	-3.9%	-2.0%	-2.1%	-4.4%	-7.7%	-5.9%	-2.0%	-3.5%	-6.8%	-3.6%	-
Male	2007	-1.6%	-2.0%	-0.7%	-3.2%	-5.0%	-1.7%	-3.0%	1.7%	-4.7%	3.0%	-
Male	2008	-0.2%	-0.3%	-1.0%	0.3%	-3.5%	-0.3%	-2.5%	10.6%	-1.5%	-3.0%	-
Male	2009	-3.2%	-3.0%	-2.3%	-4.0%	-6.5%	-6.8%	-2.7%	-0.3%	-2.1%	2.4%	-
Male	2010	3.2%	1.3%	1.0%	3.0%	0.0%	4.8%	-0.3%	16.5%	5.5%	-5.7%	-
Male	2011	0.4%	-2.2%	-1.7%	-0.4%	-4.1%	-0.3%	0.2%	8.5%	25.7%	-5.4%	-
Male	2012	-2.7%	-2.0%	-1.8%	-1.5%	-5.2%	-4.8%	-4.0%	12.1%	-24.4%	-6.7%	-
Male	2013	-2.6%	-6.6%	-1.6%	-5.0%	-6.7%	-4.5%	-1.4%	6.9%	-5.3%	-1.6%	-
Male	2014	-2.6%	-3.7%	-1.7%	-2.2%	-6.7%	-6.6%	-4.5%	3.5%	-4.4%	-7.2%	-
Male	2015	-1.0%	-2.4%	-1.2%	-2.4%	-4.7%	-1.3%	-1.6%	8.8%	-3.4%	-4.3%	-
Male	2016	-1.8%	-4.0%	-2.0%	-1.8%	-5.1%	-4.8%	0.3%	3.4%	-2.1%	-9.8%	-
Male	2017	-0.6%	-2.5%	-2.2%	0.2%	-2.4%	-21.2%	7.6%	5.2%	3.2%	-2.2%	-
Male	2018	-1.8%	-5.6%	-2.9%	-1.5%	-4.8%	-6.4%	0.9%	2.4%	-0.6%	-3.5%	-
Male	2019	-1.7%	-4.1%	-1.3%	-3.0%	-4.1%	-2.6%	-0.7%	5.2%	-7.5%	-1.2%	-
Male	2020	-1.7%	-7.3%	-0.7%	-0.7%	-4.4%	-17.8%	2.9%	8.2%	-2.6%	-0.4%	-
Male	2021	2.1%	-3.0%	-1.0%	2.0%	-0.1%	-9.1%	0.2%	8.9%	-1.4%	-0.7%	356.2%
Male	2022	6.0%	5.4%	-1.4%	6.1%	0.7%	-2.2%	5.0%	15.7%	9.2%	6.5%	166.4%
Female	1995	-	-	-	-	-	-	-	-	-	-	-
Female	1996	-7.8%	-10.5%	-0.3%	-5.1%	-9.0%	-17.6%	-7.0%	-9.0%	-19.8%	-1.0%	-
Female	1997	-2.7%	-1.2%	-1.5%	-3.2%	-6.2%	6.0%	-4.4%	-5.0%	-3.6%	1.6%	-

Female	1998	-2.7%	0.7%	0.0%	-2.7%	-6.0%	-3.7%	-7.9%	-7.8%	-1.1%	20.8%	-
Female	1999	1.6%	5.1%	0.2%	2.4%	-4.0%	12.0%	0.1%	0.4%	1.0%	-6.2%	-
Female	2000	-7.0%	-4.6%	-1.8%	-7.6%	-9.0%	-13.6%	-5.1%	-12.8%	-4.6%	-6.1%	-
Female	2001	-3.2%	-2.0%	-0.6%	-3.9%	-5.7%	-7.1%	-4.8%	-2.0%	-2.5%	-5.5%	-
Female	2002	-3.4%	-3.7%	-1.9%	-2.6%	-6.0%	-4.0%	-3.1%	-6.0%	-4.5%	-1.7%	-
Female	2003	-0.7%	2.3%	-1.2%	0.3%	-3.6%	3.8%	-1.1%	-3.9%	-0.5%	3.5%	-
Female	2004	-2.5%	1.1%	0.7%	-3.9%	-6.3%	-5.6%	-0.2%	-3.5%	-4.8%	-6.1%	-
Female	2005	0.8%	6.9%	-1.8%	2.6%	-3.0%	5.9%	1.3%	1.0%	1.2%	-1.1%	-
Female	2006	-3.3%	0.5%	-1.2%	-3.5%	-7.6%	-4.0%	-3.7%	-1.3%	-5.5%	1.8%	-
Female	2007	-1.7%	-1.5%	-1.0%	-2.2%	-5.7%	-3.5%	-0.5%	3.6%	-0.7%	3.5%	-
Female	2008	-0.3%	-0.5%	-0.4%	-0.8%	-4.2%	-0.3%	0.5%	9.9%	-2.8%	-2.5%	-
Female	2009	-3.6%	-2.2%	-1.6%	-4.5%	-8.0%	-7.7%	-5.2%	1.9%	-4.2%	-2.0%	-
Female	2010	2.4%	0.9%	0.3%	1.9%	-3.1%	2.4%	-0.7%	14.9%	7.3%	-0.9%	-
Female	2011	2.3%	-0.7%	-0.8%	-1.0%	-2.3%	0.7%	0.4%	8.8%	56.7%	5.5%	-
Female	2012	-2.9%	-0.8%	-1.2%	-1.8%	-5.6%	-5.3%	-6.1%	10.2%	-37.9%	-11.7%	-
Female	2013	-1.7%	-6.6%	-0.7%	-4.2%	-5.5%	-5.3%	-1.2%	10.1%	-7.1%	-0.8%	-
Female	2014	-2.5%	-2.7%	-0.7%	-4.0%	-6.9%	-6.5%	-0.5%	3.5%	-3.1%	-4.7%	-
Female	2015	-0.8%	-4.8%	-1.1%	-3.4%	-4.0%	-2.0%	-1.7%	8.6%	-4.0%	-8.1%	-
Female	2016	-2.0%	-2.7%	-0.5%	-3.4%	-6.4%	-7.0%	-1.4%	3.1%	-2.7%	-7.9%	-
Female	2017	-0.9%	-5.7%	-2.4%	-0.4%	-3.3%	-22.6%	4.0%	3.4%	2.5%	-3.8%	-
Female	2018	-1.0%	-5.3%	-0.5%	-2.0%	-4.4%	-6.4%	-1.3%	2.7%	-0.6%	0.8%	-
Female	2019	-1.4%	-5.9%	-1.0%	-3.7%	-4.6%	-3.2%	-1.4%	5.9%	-7.2%	-6.3%	-
Female	2020	-3.2%	-8.6%	-0.6%	-5.6%	-5.8%	-22.8%	-0.3%	3.5%	-5.9%	16.6%	-

eTable 3. Changes in crude mortality by 5-year groups (per 100,000 persons)

	Crude mortality (per 100,000 persons)						% changes between				
	2017	2018	2019	2020	2021	2022	2017–2018	2018–2019	2019–2020	2020–2021	2021–2022
Both											
Total	1075.5	1096.8	1116.2	1112.5	1172.7	1285.8	2.0%	1.8%	-0.3%	5.4%	9.6%
0–4 years	50.8	50.2	49.6	44.4	43.7	44.5	-1.0%	-1.4%	-10.4%	-1.5%	1.7%
5-9 years	6.8	7.1	7.5	6.1	6.7	6.4	4.9%	6.3%	-19.4%	9.6%	-3.9%
10–14 years	8.1	8.7	8.0	8.0	8.3	8.1	6.8%	-7.2%	-0.4%	3.9%	-3.3%
15–19 years	19.7	19.7	20.6	22.5	21.9	23.3	0.1%	4.7%	8.9%	-2.7%	6.8%
20–24 years	34.2	33.6	34.3	36.8	37.1	36.8	-1.6%	1.8%	7.6%	0.6%	-0.8%
25–29 years	38.0	37.6	35.1	37.8	39.0	38.0	-0.9%	-6.8%	7.8%	3.3%	-2.6%
30–34 years	47.3	46.5	46.0	45.6	46.1	46.4	-1.6%	-1.2%	-0.8%	1.2%	0.6%
35–39 years	61.8	61.6	61.1	60.8	60.6	63.6	-0.4%	-0.8%	-0.4%	-0.4%	5.0%
40–44 years	95.0	93.4	91.5	93.0	90.0	94.3	-1.7%	-2.1%	1.6%	-3.2%	4.8%
45–49 years	150.8	147.3	145.5	145.8	143.3	149.2	-2.3%	-1.2%	0.2%	-1.7%	4.2%
50–54 years	237.4	236.9	231.9	231.2	230.7	235.5	-0.2%	-2.1%	-0.3%	-0.2%	2.1%
55–59 years	366.9	362.5	360.1	352.4	361.5	361.0	-1.2%	-0.7%	-2.1%	2.6%	-0.2%
60–64 years	580.5	576.3	565.7	551.0	549.3	571.3	-0.7%	-1.8%	-2.6%	-0.3%	4.0%
65–69 years	936.8	927.3	918.6	893.1	891.4	920.6	-1.0%	-0.9%	-2.8%	-0.2%	3.3%
70–74 years	1415.4	1397.5	1364.6	1357.8	1411.8	1522.4	-1.3%	-2.4%	-0.5%	4.0%	7.8%
75–79 years	2321.7	2292.5	2270.8	2305.9	2378.8	2433.4	-1.3%	-0.9%	1.5%	3.2%	2.3%
80–84 years	4286.4	4223.8	4186.8	4023.1	4064.7	4327.5	-1.5%	-0.9%	-3.9%	1.0%	6.5%
85–89 years	7972.5	7893.2	7743.9	7411.0	7574.5	8133.8	-1.0%	-1.9%	-4.3%	2.2%	7.4%
90–94 years	14149.8	14042.8	13965.7	13574.6	13904.4	14913.5	-0.8%	-0.5%	-2.8%	2.4%	7.3%
95 years and over	28006.2	27073.4	26646.3	25811.9	27101.0	30192.9	-3.3%	-1.6%	-3.1%	5.0%	11.4%
Men											
Total	1138.5	1156.5	1175.0	1178.0	1236.7	1347.8	1.6%	1.6%	0.3%	5.0%	9.0%
0–4 years	52.4	51.9	51.3	47.1	46.2	46.7	-0.9%	-1.2%	-8.1%	-1.9%	1.0%
5–9 years	7.9	7.9	8.1	5.9	7.6	6.7	0.0%	3.3%	-27.9%	30.5%	-12.3%
10–14 years	10.0	9.7	8.7	9.0	9.0	8.7	-2.8%	-10.6%	3.7%	-0.4%	-3.5%
15–19 years	26.7	25.1	26.3	28.3	26.7	27.9	-6.2%	5.1%	7.5%	-5.5%	4.4%
20–24 years	48.3	45.9	46.7	49.2	47.9	46.9	-5.1%	1.7%	5.5%	-2.7%	-2.1%
25–29 years	50.5	50.4	45.7	49.1	50.3	47.9	-0.2%	-9.3%	7.3%	2.5%	-4.7%
30–34 years	61.5	60.6	59.4	60.4	58.1	58.0	-1.5%	-1.9%	1.7%	-3.9%	-0.2%
35–39 years	78.7	77.7	76.6	75.2	76.9	78.0	-1.3%	-1.4%	-1.8%	2.3%	1.4%
40–44 years	116.7	115.5	112.6	114.7	109.8	116.0	-1.0%	-2.5%	1.8%	-4.2%	5.6%
45–49 years	189.6	181.7	179.9	180.9	179.7	184.0	-4.2%	-0.9%	0.6%	-0.7%	2.4%
50–54 years	305.0	303.1	297.2	293.1	294.1	300.3	-0.6%	-1.9%	-1.4%	0.3%	2.1%
55–59 years	493.7	484.5	479.7	472.6	484.4	481.3	-1.9%	-1.0%	-1.5%	2.5%	-0.6%
60–64 years	814.9	807.7	793.3	770.7	766.7	792.3	-0.9%	-1.8%	-2.8%	-0.5%	3.3%
65–69 years	1346.1	1330.0	1317.9	1284.0	1274.6	1316.4	-1.2%	-0.9%	-2.6%	-0.7%	3.3%
70–74 years	2056.7	2020.5	1977.0	1967.8	2049.2	2214.5	-1.8%	-2.2%	-0.5%	4.1%	8.1%
75–79 years	3323.0	3274.7	3259.5	3332.8	3435.6	3506.7	-1.5%	-0.5%	2.3%	3.1%	2.1%
80–84 years	6041.7	5926.1	5864.8	5661.4	5739.5	6072.8	-1.9%	-1.0%	-3.5%	1.4%	5.8%
85–89 years	11086.9	10912.7	10653.7	10286.6	10440.5	11199.6	-1.6%	-2.4%	-3.4%	1.5%	7.3%
90–94 years	18838.9	18580.7	18383.6	18061.4	18473.7	19811.3	-1.4%	-1.1%	-1.8%	2.3%	7.2%
95 years and over	34097.5	32714.1	31145.8	30779.8	32526.3	36611.6	-4.1%	-4.8%	-1.2%	5.7%	12.6%
Women											
Total	1015.7	1040.3	1060.5	1050.4	1112.2	1227.1	2.4%	1.9%	-1.0%	5.9%	10.3%
0–4 years	49.1	48.5	47.8	41.5	41.1	42.1	-1.1%	-1.5%	-13.1%	-1.0%	2.4%
5–9 years	5.6	6.3	6.9	6.3	5.6	6.1	12.0%	10.2%	-8.9%	-10.9%	7.9%
10–14 years	6.1	7.6	7.4	6.9	7.6	7.4	23.3%	-2.7%	-5.6%	9.9%	-3.1%

15–19 years	12.2	14.0	14.6	16.3	16.7	18.5	14.6%	4.1%	11.6%	2.5%	10.7%
20–24 years	19.3	20.7	21.1	24.0	25.8	26.1	7.2%	2.1%	13.5%	7.5%	1.5%
25–29 years	24.8	24.2	23.9	26.0	27.3	27.7	-2.5%	-1.5%	8.9%	5.0%	1.5%
30–34 years	32.6	32.0	32.0	30.2	33.7	34.3	-1.8%	0.3%	-5.8%	11.7%	1.9%
35–39 years	44.4	45.0	45.1	46.0	43.7	48.7	1.4%	0.3%	1.9%	-4.9%	11.6%
40–44 years	72.6	70.5	69.6	70.5	69.4	71.8	-2.9%	-1.3%	1.2%	-1.5%	3.5%
45–49 years	110.8	111.9	110.0	109.5	105.7	113.3	1.0%	-1.7%	-0.4%	-3.5%	7.2%
50–54 years	168.6	169.4	165.3	168.0	165.9	169.1	0.5%	-2.5%	1.7%	-1.2%	1.9%
55–59 years	240.3	240.3	240.1	231.7	238.0	239.9	0.0%	-0.1%	-3.5%	2.7%	0.8%
60–64 years	352.1	350.6	343.2	335.7	335.9	353.8	-0.4%	-2.1%	-2.2%	0.1%	5.3%
65–69 years	553.3	549.9	543.5	523.8	528.4	544.9	-0.6%	-1.2%	-3.6%	0.9%	3.1%
70–74 years	850.5	845.3	818.3	812.5	841.9	904.0	-0.6%	-3.2%	-0.7%	3.6%	7.4%
75–79 years	1513.6	1497.2	1470.9	1481.2	1529.3	1561.2	-1.1%	-1.8%	0.7%	3.2%	2.1%
80–84 years	3079.2	3039.3	3008.0	2869.9	2874.5	3080.6	-1.3%	-1.0%	-4.6%	0.2%	7.2%
85–89 years	6328.2	6276.6	6158.1	5835.3	5973.6	6398.0	-0.8%	-1.9%	-5.2%	2.4%	7.1%
90–94 years	12472.6	12355.2	12301.6	11867.8	12150.1	12992.1	-0.9%	-0.4%	-3.5%	2.4%	6.9%
95 years and over	26710.4	26001.4	25631.6	24738.2	25881.1	28716.3	-2.7%	-1.4%	-3.5%	4.6%	11.0%

*Increasing mortality (plus percent change) was shown in red.

eTable 4. Changes in cause-specific truncated age-standardized mortality (per 100,000 persons)

	Age-standardized mortality (per 100,000 persons)						% changes between				
	2017	2018	2019	2020	2021	2022	2017-2018	2018-2019	2019-2020	2020-2021	2021-2022
Both											
All-cause											
0–39 years	34.6	34.4	34.2	34.4	34.5	35.0	-0.5%	-0.8%	0.6%	0.3%	1.5%
40–69 years	410.8	406.7	401.5	393.2	393.2	404.6	-1.0%	-1.3%	-2.1%	0.0%	2.9%
70 years and over	4361.6	4302.1	4246.4	4137.9	4248.2	4542.9	-1.4%	-1.3%	-2.6%	2.7%	6.9%
Malignant neoplasms											
0–39 years	5.3	5.0	5.1	4.8	4.8	4.9	-4.5%	1.0%	-5.4%	0.5%	1.6%
40–69 years	184.6	179.5	175.4	169.7	166.1	164.4	-2.8%	-2.3%	-3.2%	-2.2%	-1.0%
70 years and over	1092.7	1075.2	1068.3	1069.9	1068.7	1066.0	-1.6%	-0.6%	0.1%	-0.1%	-0.2%
Heart diseases											
0–39 years	2.1	2.0	2.1	2.0	2.0	2.1	-4.5%	2.3%	-2.0%	-0.4%	2.5%
40–69 years	49.7	49.8	49.7	48.6	49.0	51.0	0.2%	-0.2%	-2.4%	0.8%	4.2%
70 years and over	697.6	684.4	660.1	638.6	648.2	687.3	-1.9%	-3.5%	-3.3%	1.5%	6.0%
Cerebrovascular diseases											
0–39 years	1.0	1.1	1.1	1.0	1.0	1.0	9.3%	-3.9%	-7.8%	-0.7%	-2.4%
40–69 years	30.1	29.2	28.8	28.2	27.5	28.2	-2.9%	-1.4%	-2.1%	-2.7%	2.5%
70 years and over	367.4	349.8	333.8	314.9	312.7	313.4	-4.8%	-4.6%	-5.7%	-0.7%	0.2%
Pneumonia & Bronchitis											
0–39 years	0.3	0.3	0.3	0.3	0.2	0.2	-2.3%	7.1%	-12.0%	-25.1%	7.1%
40–69 years	8.8	9.2	9.2	7.9	6.9	6.7	3.8%	0.5%	-14.4%	-13.2%	-1.7%
70 years and over	359.1	336.3	327.5	261.8	237.2	232.9	-6.4%	-2.6%	-20.1%	-9.4%	-1.8%
Suicide											
0–39 years	11.1	11.1	11.1	12.4	12.7	12.6	0.2%	-0.8%	12.3%	2.7%	-0.8%
40–69 years	19.8	19.1	18.7	18.9	19.0	20.7	-3.3%	-2.2%	1.0%	0.6%	8.9%
70 years and over	19.7	19.2	18.0	18.4	17.8	18.6	-2.2%	-6.3%	1.9%	-2.8%	4.4%
COVID-19											
0–39 years	-	-	-	0.0	0.2	0.4	-	-	-	1261.8%	135.9%
40–69 years	-	-	-	0.9	5.9	5.9	-	-	-	569.7%	0.3%
70 years and over	-	-	-	10.9	48.4	146.6	-	-	-	345.0%	202.7%
Men											
All-cause											
0–39 years	44.0	43.2	42.4	42.8	42.6	42.3	-2.0%	-1.7%	0.9%	-0.6%	-0.6%
40–69 years	568.0	560.5	553.3	541.8	540.7	554.8	-1.3%	-1.3%	-2.1%	-0.2%	2.6%
70 years and over	5995.7	5884.7	5780.3	5681.4	5827.9	6223.5	-1.9%	-1.8%	-1.7%	2.6%	6.8%
Malignant neoplasms											
0–39 years	4.8	4.5	4.4	4.5	4.5	4.1	-4.9%	-1.9%	1.0%	0.5%	-9.1%
40–69 years	230.3	220.7	214.1	206.4	200.2	194.9	-4.2%	-3.0%	-3.6%	-3.0%	-2.6%
70 years and over	1661.5	1619.3	1606.4	1607.3	1600.2	1584.4	-2.5%	-0.8%	0.1%	-0.4%	-1.0%
Heart diseases											
0–39 years	3.1	3.0	3.0	3.1	3.0	3.0	-4.4%	-0.3%	3.5%	-2.9%	0.2%
40–69 years	78.8	78.7	78.8	78.0	78.5	81.6	-0.1%	0.0%	-0.9%	0.6%	3.9%
70 years and over	869.7	854.7	823.5	817.9	837.0	892.1	-1.7%	-3.6%	-0.7%	2.3%	6.6%
Cerebrovascular diseases											
0–39 years	1.4	1.4	1.4	1.3	1.3	1.3	1.5%	-2.0%	-5.6%	-0.7%	-0.2%
40–69 years	42.7	41.4	41.3	39.8	39.3	40.5	-3.0%	-0.3%	-3.7%	-1.2%	3.2%
70 years and over	465.9	442.0	420.5	401.4	402.1	402.8	-5.1%	-4.9%	-4.5%	0.2%	0.2%
Pneumonia & Bronchitis											

0–39 years	0.3	0.3	0.4	0.3	0.2	0.2	-1.1%	5.1%	-5.7%	-35.1%	11.5%
40–69 years	14.0	14.7	14.4	12.7	10.9	10.7	5.3%	-2.5%	-11.5%	-14.6%	-1.3%
70 years and over	596.6	554.6	540.4	442.1	403.7	394.3	-7.0%	-2.6%	-18.2%	-8.7%	-2.3%
Suicide											
0–39 years	16.0	15.7	15.6	16.5	16.6	16.6	-1.8%	-0.6%	6.0%	0.6%	-0.3%
40–69 years	28.6	27.2	27.2	26.0	26.1	28.9	-4.9%	0.1%	-4.4%	0.2%	10.8%
70 years and over	29.9	29.2	27.9	28.0	26.9	28.6	-2.2%	-4.6%	0.5%	-4.0%	6.4%
COVID-19											
0–39 years	-	-	-	0.0	0.3	0.5	-	-	-	1157.7%	83.8%
40–69 years	-	-	-	1.5	9.3	8.9	-	-	-	527.2%	-3.8%
70 years and over	-	-	-	16.8	70.9	223.3	-	-	-	321.8%	214.8%
Women											
All-cause											
0–39 years	24.8	25.3	25.5	25.6	26.1	27.4	2.1%	0.8%	0.1%	2.0%	5.0%
40–69 years	258.9	258.0	254.4	248.6	249.4	258.0	-0.3%	-1.4%	-2.3%	0.3%	3.4%
70 years and over	3381.3	3340.7	3293.3	3181.2	3260.8	3489.6	-1.2%	-1.4%	-3.4%	2.5%	7.0%
Malignant neoplasms											
0–39 years	5.8	5.5	5.7	5.1	5.2	5.7	-4.2%	3.4%	-10.5%	0.5%	11.4%
40–69 years	141.2	140.5	138.7	134.8	133.6	135.3	-0.5%	-1.2%	-2.8%	-0.9%	1.3%
70 years and over	732.2	728.5	721.7	724.8	723.0	728.0	-0.5%	-0.9%	0.4%	-0.2%	0.7%
Heart diseases											
0–39 years	1.2	1.1	1.2	1.0	1.1	1.2	-4.9%	9.6%	-16.3%	7.3%	8.9%
40–69 years	21.4	21.7	21.4	19.7	20.0	21.0	1.0%	-1.1%	-8.1%	1.4%	5.1%
70 years and over	591.2	577.9	554.9	525.5	529.9	557.3	-2.2%	-4.0%	-5.3%	0.8%	5.2%
Cerebrovascular diseases											
0–39 years	0.6	0.8	0.8	0.7	0.7	0.6	26.5%	-7.3%	-12.0%	-0.9%	-7.0%
40–69 years	17.8	17.4	16.7	17.0	15.9	16.0	-2.7%	-3.9%	1.7%	-6.2%	0.8%
70 years and over	303.3	288.8	275.1	256.7	252.1	252.4	-4.8%	-4.7%	-6.7%	-1.8%	0.1%
Pneumonia & Bronchitis											
0–39 years	0.3	0.2	0.3	0.2	0.2	0.2	-4.3%	10.1%	-20.9%	-8.3%	1.8%
40–69 years	3.9	3.8	4.3	3.2	3.0	2.9	-1.9%	11.4%	-24.3%	-8.2%	-3.0%
70 years and over	240.5	224.7	216.2	167.1	148.9	145.8	-6.6%	-3.8%	-22.7%	-10.9%	-2.1%
Suicide											
0–39 years	6.0	6.4	6.3	8.1	8.7	8.5	5.9%	-1.4%	28.5%	7.0%	-1.9%
40–69 years	11.0	11.0	10.2	11.7	11.9	12.5	0.7%	-7.9%	15.2%	1.5%	4.6%
70 years and over	12.9	12.4	11.3	11.9	11.8	11.9	-4.1%	-8.7%	5.4%	-1.3%	1.5%
COVID-19											
0–39 years	-	-	-	0.0	0.1	0.3	-	-	-	1816.3%	315.9%
40–69 years	-	-	-	0.3	2.5	2.9	-	-	-	762.4%	16.6%
70 years and over	-	-	-	7.1	34.4	102.8	-	-	-	385.8%	198.5%

COVID-19, coronavirus disease 2019.

*Increasing mortality (plus percent change) was shown in red.

eTable 5. Age-standardized mortality rate by prefectures calculated using the 2015 Japanese Standard population (per 100,000 persons, rounding 85 years and over)

			Age-standardized mortality rate (rounding 85 years and over)						% changes between				
Sex	Prefecture number	Prefecture	2017	2018	2019	2020	2021	2022	2017–2018	2018–2019	2019–2020	2020–2021	2021–2022
Both	0	Total	1018.5	1008.3	998.6	975.3	999.7	1064.6	-1.0%	-1.0%	-2.3%	2.5%	6.5%
Both	1	Hokkaido	1013.6	1016.3	1013.3	992.9	1034.6	1092.7	0.3%	-0.3%	-2.0%	4.2%	5.6%
Both	2	Aomori	1127.6	1130.4	1141.5	1097.3	1131.3	1189.6	0.2%	1.0%	-3.9%	3.1%	5.2%
Both	3	Iwate	1071.8	1067.2	1071.6	1027.6	1034.6	1126.6	-0.4%	0.4%	-4.1%	0.7%	8.9%
Both	4	Miyagi	986.8	981.3	982.5	963.9	994.2	1055.7	-0.6%	0.1%	-1.9%	3.1%	6.2%
Both	5	Akita	1072.3	1055.4	1062.3	1031.8	1061.5	1131.8	-1.6%	0.7%	-2.9%	2.9%	6.6%
Both	6	Yamagata	1023.8	1011.0	1026.9	1001.7	1020.8	1081.7	-1.2%	1.6%	-2.5%	1.9%	6.0%
Both	7	Fukushima	1088.0	1069.6	1070.3	1047.8	1081.7	1152.3	-1.7%	0.1%	-2.1%	3.2%	6.5%
Both	8	Ibaraki	1083.3	1079.1	1075.8	1040.0	1044.5	1121.8	-0.4%	-0.3%	-3.3%	0.4%	7.4%
Both	9	Tochigi	1100.6	1081.3	1076.7	1030.4	1057.7	1140.0	-1.8%	-0.4%	-4.3%	2.7%	7.8%
Both	10	Gunma	1059.7	1052.1	1044.6	1031.8	1056.4	1135.6	-0.7%	-0.7%	-1.2%	2.4%	7.5%
Both	11	Saitama	1030.5	1017.7	1005.6	988.7	1010.8	1066.5	-1.2%	-1.2%	-1.7%	2.2%	5.5%
Both	12	Chiba	1014.3	986.0	991.9	974.6	989.4	1061.0	-2.8%	0.6%	-1.7%	1.5%	7.2%
Both	13	Tokyo	984.1	975.8	962.5	945.5	969.4	1032.8	-0.8%	-1.4%	-1.8%	2.5%	6.5%
Both	14	Kanagawa	985.2	974.2	962.1	929.5	953.3	1017.6	-1.1%	-1.2%	-3.4%	2.6%	6.7%
Both	15	Niigata	1023.7	1033.4	1038.5	997.6	1038.8	1069.2	0.9%	0.5%	-3.9%	4.1%	2.9%
Both	16	Yamanashi	1018.2	985.9	984.0	960.8	995.3	1070.4	-3.2%	-0.2%	-2.4%	3.6%	7.5%
Both	17	Nagano	1006.4	982.8	973.6	954.5	976.6	1035.3	-2.3%	-0.9%	-2.0%	2.3%	6.0%
Both	18	Toyama	992.2	969.3	992.2	960.9	992.4	1055.1	-2.3%	2.4%	-3.2%	3.3%	6.3%
Both	19	Ishikawa	991.6	989.1	1000.2	966.0	988.6	1058.8	-0.3%	1.1%	-3.4%	2.3%	7.1%
Both	20	Fukui	962.4	935.4	940.9	916.8	923.0	995.0	-2.8%	0.6%	-2.6%	0.7%	7.8%
Both	21	Gifu	1043.2	1019.3	1016.1	965.4	1010.6	1071.7	-2.3%	-0.3%	-5.0%	4.7%	6.0%
Both	22	Shizuoka	1027.2	1023.0	1008.2	992.2	991.5	1064.4	-0.4%	-1.4%	-1.6%	-0.1%	7.4%
Both	23	Aichi	1025.7	1017.5	1000.9	979.6	994.5	1065.1	-0.8%	-1.6%	-2.1%	1.5%	7.1%
Both	24	Mie	1033.2	1028.6	1004.2	990.6	1014.2	1073.1	-0.4%	-2.4%	-1.3%	2.4%	5.8%
Both	25	Shiga	973.2	969.8	933.7	910.5	925.0	999.5	-0.3%	-3.7%	-2.5%	1.6%	8.1%
Both	26	Kyoto	972.0	949.3	939.5	914.3	935.0	1018.3	-2.3%	-1.0%	-2.7%	2.3%	8.9%
Both	27	Osaka	1045.3	1037.9	1016.1	1000.6	1031.9	1093.3	-0.7%	-2.1%	-1.5%	3.1%	5.9%
Both	28	Hyogo	994.4	979.3	962.1	949.1	978.1	1023.4	-1.5%	-1.8%	-1.3%	3.1%	4.6%
Both	29	Nara	977.4	968.6	939.8	918.6	939.0	1009.0	-0.9%	-3.0%	-2.2%	2.2%	7.4%
Both	30	Wakayama	1079.3	1083.0	1053.5	1016.6	1026.5	1115.0	0.3%	-2.7%	-3.5%	1.0%	8.6%
Both	31	Tottori	1035.1	986.9	1030.7	948.9	991.3	1045.4	-4.7%	4.4%	-7.9%	4.5%	5.5%
Both	32	Shimane	990.5	980.7	973.1	964.0	983.3	1027.3	-1.0%	-0.8%	-0.9%	2.0%	4.5%
Both	33	Oakayama	968.2	982.8	950.4	933.5	961.0	1027.6	1.5%	-3.3%	-1.8%	3.0%	6.9%
Both	34	Hiroshima	997.8	997.1	976.6	931.7	954.2	1026.7	-0.1%	-2.1%	-4.6%	2.4%	7.6%
Both	35	Yamaguchi	1042.9	1032.3	1029.8	993.2	1023.1	1073.8	-1.0%	-0.2%	-3.6%	3.0%	5.0%
Both	36	Tokushima	1061.6	1018.2	1015.8	993.7	1031.5	1073.0	-4.1%	-0.2%	-2.2%	3.8%	4.0%
Both	37	Kagawa	997.3	1002.5	989.6	995.9	986.7	1072.7	0.5%	-1.3%	0.6%	-0.9%	8.7%
Both	38	Ehime	1049.4	1040.3	1029.8	1004.4	1034.4	1079.2	-0.9%	-1.0%	-2.5%	3.0%	4.3%
Both	39	Kochi	1014.1	1014.4	1011.7	975.7	1011.1	1093.3	0.0%	-0.3%	-3.6%	3.6%	8.1%
Both	40	Fukuoka	1010.3	999.5	990.5	958.6	992.0	1053.8	-1.1%	-0.9%	-3.2%	3.5%	6.2%
Both	41	Saga	1018.7	1019.6	993.5	984.3	998.1	1075.0	0.1%	-2.6%	-0.9%	1.4%	7.7%
Both	42	Nagasaki	1034.1	1030.8	1010.7	1010.0	1030.6	1074.6	-0.3%	-2.0%	-0.1%	2.0%	4.3%
Both	43	Kumamoto	981.9	955.1	954.7	925.1	948.9	1037.5	-2.7%	0.0%	-3.1%	2.6%	9.3%
Both	44	Oita	979.2	969.7	959.5	942.4	971.6	1033.3	-1.0%	-1.1%	-1.8%	3.1%	6.4%
Both	45	Miyazaki	1017.3	1010.7	980.9	995.3	1005.7	1095.4	-0.7%	-2.9%	1.5%	1.0%	8.9%
Both	46	Kagoshima	1041.6	1040.1	1017.8	999.6	1015.6	1088.2	-0.1%	-2.1%	-1.8%	1.6%	7.1%
Both	47	Okinawa	996.4	977.3	983.5	944.8	1020.5	1094.6	-1.9%	0.6%	-3.9%	8.0%	7.3%
Male	0	Total	1350.5	1330.4	1313.2	1290.3	1318.4	1398.3	-1.5%	-1.3%	-1.7%	2.2%	6.1%
Male	1	Hokkaido	1365.8	1361.5	1351.1	1316.9	1370.6	1442.3	-0.3%	-0.8%	-2.5%	4.1%	5.2%
Male	2	Aomori	1578.4	1558.1	1589.8	1506.8	1530.3	1620.5	-1.3%	2.0%	-5.2%	1.6%	5.9%
Male	3	Iwate	1467.5	1439.5	1445.6	1366.6	1383.3	1492.5	-1.9%	0.4%	-5.5%	1.2%	7.9%
Male	4	Miyagi	1303.3	1287.6	1294.3	1263.9	1290.0	1364.5	-1.2%	0.5%	-2.3%	2.1%	5.8%
Male	5	Akita	1457.3	1436.7	1429.5	1433.5	1433.9	1522.6	-1.4%	-0.5%	0.3%	0.0%	6.2%
Male	6	Yamagata	1358.3	1343.8	1366.3	1309.7	1330.7	1404.9	-1.1%	1.7%	-4.1%	1.6%	5.6%
Male	7	Fukushima	1448.4	1398.3	1389.1	1378.1	1416.6	1491.4	-3.5%	-0.7%	-0.8%	2.8%	5.3%
Male	8	Ibaraki	1429.4	1408.7	1391.5	1350.9	1344.3	1440.4	-1.4%	-1.2%	-2.9%	-0.5%	7.1%
Male	9	Tochigi	1426.8	1403.7	1361.9	1325.6	1371.1	1461.1	-1.6%	-3.0%	-2.7%	3.4%	6.6%
Male	10	Gunma	1383.3	1360.2	1349.3	1347.6	1392.5	1463.8	-1.7%	-0.8%	-0.1%	3.3%	5.1%
Male	11	Saitama	1329.1	1323.0	1306.3	1279.0	1316.6	1375.5	-0.5%	-1.3%	-2.1%	2.9%	4.5%
Male	12	Chiba	1316.2	1273.8	1275.5	1271.7	1285.5	1375.1	-3.2%	0.1%	-0.3%	1.1%	7.0%
Male	13	Tokyo	1313.6	1295.1	1272.9	1261.3	1292.7	1372.8	-1.4%	-1.7%	-0.9%	2.5%	6.2%

Male	14	Kanagawa	1293.5	1263.4	1248.7	1219.2	1242.7	1338.8	-2.3%	-1.2%	-2.4%	1.9%	7.7%
Male	15	Niigata	1376.9	1377.6	1376.0	1330.0	1385.6	1401.9	0.1%	-0.1%	-3.3%	4.2%	1.2%
Male	16	Yamanashi	1355.3	1356.1	1324.7	1282.3	1343.2	1445.1	0.1%	-2.3%	-3.2%	4.8%	7.6%
Male	17	Nagano	1341.9	1292.7	1314.8	1289.1	1292.3	1356.5	-3.7%	1.7%	-2.0%	0.2%	5.0%
Male	18	Toyama	1331.6	1277.9	1303.3	1283.9	1310.9	1354.8	-4.0%	2.0%	-1.5%	2.1%	3.3%
Male	19	Ishikawa	1296.6	1310.0	1303.2	1268.6	1290.1	1337.7	1.0%	-0.5%	-2.7%	1.7%	3.7%
Male	20	Fukui	1240.2	1207.4	1208.5	1185.1	1174.3	1288.3	-2.6%	0.1%	-1.9%	-0.9%	9.7%
Male	21	Gifu	1368.3	1337.8	1319.2	1249.5	1324.2	1398.1	-2.2%	-1.4%	-5.3%	6.0%	5.6%
Male	22	Shizuoka	1353.2	1333.2	1313.2	1299.8	1292.1	1377.4	-1.5%	-1.5%	-1.0%	-0.6%	6.6%
Male	23	Aichi	1346.3	1332.7	1303.8	1285.3	1293.4	1395.4	-1.0%	-2.2%	-1.4%	0.6%	7.9%
Male	24	Mie	1338.1	1330.0	1315.0	1285.3	1326.0	1385.7	-0.6%	-1.1%	-2.3%	3.2%	4.5%
Male	25	Shiga	1282.8	1253.3	1202.0	1176.5	1185.1	1274.3	-2.3%	-4.1%	-2.1%	0.7%	7.5%
Male	26	Kyoto	1277.0	1256.6	1223.5	1210.9	1226.0	1340.6	-1.6%	-2.6%	-1.0%	1.2%	9.4%
Male	27	Osaka	1390.7	1384.0	1352.0	1350.0	1394.9	1468.5	-0.5%	-2.3%	-0.1%	3.3%	5.3%
Male	28	Hyogo	1317.1	1296.5	1259.0	1270.3	1300.2	1350.4	-1.6%	-2.9%	0.9%	2.4%	3.9%
Male	29	Nara	1264.5	1248.4	1221.7	1198.2	1205.4	1290.2	-1.3%	-2.1%	-1.9%	0.6%	7.0%
Male	30	Wakayama	1427.1	1453.9	1404.1	1355.8	1393.2	1506.9	1.9%	-3.4%	-3.4%	2.8%	8.2%
Male	31	Tottori	1400.0	1375.2	1337.2	1293.8	1329.7	1406.0	-1.8%	-2.8%	-3.2%	2.8%	5.7%
Male	32	Shimane	1305.5	1308.8	1310.7	1279.2	1341.5	1370.2	0.3%	0.1%	-2.4%	4.9%	2.1%
Male	33	Oakayama	1286.1	1298.3	1264.3	1249.8	1271.2	1353.1	1.0%	-2.6%	-1.1%	1.7%	6.4%
Male	34	Hiroshima	1321.7	1332.7	1290.9	1239.0	1250.4	1356.3	0.8%	-3.1%	-4.0%	0.9%	8.5%
Male	35	Yamaguchi	1404.3	1367.3	1381.5	1343.8	1355.9	1430.3	-2.6%	1.0%	-2.7%	0.9%	5.5%
Male	36	Tokushima	1412.8	1342.5	1314.8	1301.6	1361.5	1407.1	-5.0%	-2.1%	-1.0%	4.6%	3.3%
Male	37	Kagawa	1305.9	1296.1	1262.9	1317.6	1292.4	1387.1	-0.8%	-2.6%	4.3%	-1.9%	7.3%
Male	38	Ehime	1430.0	1390.9	1354.3	1325.1	1383.8	1418.1	-2.7%	-2.6%	-2.2%	4.4%	2.5%
Male	39	Kochi	1359.9	1361.1	1358.0	1327.6	1402.0	1489.8	0.1%	-0.2%	-2.2%	5.6%	6.3%
Male	40	Fukuoka	1378.7	1351.0	1330.5	1296.2	1346.1	1412.2	-2.0%	-1.5%	-2.6%	3.8%	4.9%
Male	41	Saga	1373.3	1380.0	1301.4	1320.7	1341.2	1413.1	0.5%	-5.7%	1.5%	1.6%	5.4%
Male	42	Nagasaki	1387.0	1370.5	1360.6	1338.1	1379.5	1413.8	-1.2%	-0.7%	-1.7%	3.1%	2.5%
Male	43	Kumamoto	1296.1	1271.2	1255.8	1239.9	1252.7	1370.5	-1.9%	-1.2%	-1.3%	1.0%	9.4%
Male	44	Oita	1317.8	1289.9	1254.3	1268.0	1270.8	1378.4	-2.1%	-2.8%	1.1%	0.2%	8.5%
Male	45	Miyazaki	1370.9	1346.8	1292.7	1324.8	1321.1	1448.8	-1.8%	-4.0%	2.5%	-0.3%	9.7%
Male	46	Kagoshima	1419.4	1398.0	1350.7	1316.3	1334.9	1415.2	-1.5%	-3.4%	-2.6%	1.4%	6.0%
Male	47	Okinawa	1316.5	1244.0	1313.2	1258.3	1344.4	1421.9	-5.5%	5.6%	-4.2%	6.8%	5.8%
Female	0	Total	783.4	778.2	771.3	747.8	768.0	821.7	-0.7%	-0.9%	-3.0%	2.7%	7.0%
Female	1	Hokkaido	779.9	784.4	783.8	771.8	805.5	852.7	0.6%	-0.1%	-1.5%	4.4%	5.9%
Female	2	Aomori	852.2	853.8	849.1	824.1	859.9	918.5	0.2%	-0.6%	-2.9%	4.3%	6.8%
Female	3	Iwate	812.7	814.2	825.0	791.5	789.5	878.7	0.2%	1.3%	-4.1%	-0.2%	11.3%
Female	4	Miyagi	760.9	753.4	763.4	747.6	782.2	824.7	-1.0%	1.3%	-2.1%	4.6%	5.4%
Female	5	Akita	810.6	798.8	818.0	766.5	807.7	867.1	-1.5%	2.4%	-6.3%	5.4%	7.4%
Female	6	Yamagata	789.2	781.0	789.2	781.8	796.1	841.7	-1.0%	1.0%	-0.9%	1.8%	5.7%
Female	7	Fukushima	834.3	831.6	829.2	807.3	839.0	899.2	-0.3%	-0.3%	-2.6%	3.9%	7.2%
Female	8	Ibaraki	828.8	828.1	832.5	796.7	810.1	869.8	-0.1%	0.5%	-4.3%	1.7%	7.4%
Female	9	Tochigi	862.3	850.2	844.6	805.8	818.4	891.9	-1.4%	-0.7%	-4.6%	1.6%	9.0%
Female	10	Gunma	805.4	818.1	815.9	795.9	799.4	877.9	1.6%	-0.3%	-2.5%	0.4%	9.8%
Female	11	Saitama	803.4	785.1	774.9	762.4	773.1	823.9	-2.3%	-1.3%	-1.6%	1.4%	6.6%
Female	12	Chiba	785.8	762.3	769.0	743.6	756.9	815.7	-3.0%	0.9%	-3.3%	1.8%	7.8%
Female	13	Tokyo	750.0	747.3	737.3	718.0	736.1	789.4	-0.4%	-1.3%	-2.6%	2.5%	7.3%
Female	14	Kanagawa	758.8	756.6	744.5	711.1	732.7	776.8	-0.3%	-1.6%	-4.5%	3.0%	6.0%
Female	15	Niigata	773.9	791.2	791.6	760.9	792.1	830.3	2.2%	0.1%	-3.9%	4.1%	4.8%
Female	16	Yamanashi	773.0	755.1	754.8	743.0	756.8	825.8	-2.3%	0.0%	-1.6%	1.9%	9.1%
Female	17	Nagano	779.0	757.9	751.1	725.8	758.2	799.6	-2.7%	-0.9%	-3.4%	4.5%	5.5%
Female	18	Toyama	759.6	744.8	769.4	734.9	774.1	832.2	-2.0%	3.3%	-4.5%	5.3%	7.5%
Female	19	Ishikawa	761.3	777.4	759.5	741.3	766.1	848.8	2.1%	-2.3%	-2.4%	3.3%	10.8%
Female	20	Fukui	749.5	733.1	739.6	718.1	732.4	778.3	-2.2%	0.9%	-2.9%	2.0%	6.3%
Female	21	Gifu	810.2	795.2	797.6	761.5	784.1	823.5	-1.9%	0.3%	-4.5%	3.0%	5.0%
Female	22	Shizuoka	791.5	795.5	785.0	765.4	767.1	826.4	0.5%	-1.3%	-2.5%	0.2%	7.7%
Female	23	Aichi	789.7	787.3	774.8	752.1	768.2	817.9	-0.3%	-1.6%	-2.9%	2.1%	6.5%
Female	24	Mie	801.7	801.5	774.2	774.2	787.6	840.8	0.0%	-3.4%	0.0%	1.7%	6.8%
Female	25	Shiga	766.8	760.2	733.6	712.2	731.0	771.7	-0.9%	-3.5%	-2.9%	2.6%	5.6%
Female	26	Kyoto	761.7	740.9	734.6	703.2	726.8	789.3	-2.7%	-0.8%	-4.3%	3.4%	8.6%
Female	27	Osaka	796.0	789.4	772.5	750.4	771.0	823.2	-0.8%	-2.1%	-2.8%	2.7%	6.8%
Female	28	Hyogo	769.2	758.5	746.7	724.1	748.6	789.2	-1.4%	-1.6%	-3.0%	3.4%	5.4%
Female	29	Nara	772.1	757.3	738.6	717.6	743.0	805.5	-1.9%	-2.5%	-2.8%	3.5%	8.4%
Female	30	Wakayama	839.2	843.9	809.6	784.4	781.3	855.6	0.6%	-4.1%	-3.1%	-0.4%	9.5%

Female	31	Tottori	783.0	750.3	789.3	708.6	754.5	807.0	-4.2%	5.2%	-10.2%	6.5%	7.0%
Female	32	Shimane	765.3	759.2	730.9	738.5	738.2	780.0	-0.8%	-3.7%	1.0%	0.0%	5.7%
Female	33	Oakayama	744.3	756.7	722.4	708.4	736.9	788.1	1.7%	-4.5%	-1.9%	4.0%	6.9%
Female	34	Hiroshima	771.5	766.9	750.1	715.2	736.7	789.5	-0.6%	-2.2%	-4.6%	3.0%	7.2%
Female	35	Yamaguchi	793.8	809.8	802.7	752.8	793.0	839.0	2.0%	-0.9%	-6.2%	5.3%	5.8%
Female	36	Tokushima	822.2	784.9	802.2	775.4	791.2	828.8	-4.5%	2.2%	-3.3%	2.0%	4.8%
Female	37	Kagawa	774.3	784.1	786.8	764.8	760.9	844.1	1.3%	0.3%	-2.8%	-0.5%	10.9%
Female	38	Ehime	807.7	801.3	797.3	784.4	791.5	841.8	-0.8%	-0.5%	-1.6%	0.9%	6.4%
Female	39	Kochi	778.6	782.6	772.1	735.2	744.4	836.0	0.5%	-1.3%	-4.8%	1.3%	12.3%
Female	40	Fukuoka	773.2	768.9	766.4	735.3	758.2	811.9	-0.6%	-0.3%	-4.1%	3.1%	7.1%
Female	41	Saga	793.2	786.4	761.5	754.3	771.6	827.1	-0.9%	-3.2%	-0.9%	2.3%	7.2%
Female	42	Nagasaki	804.0	795.9	773.4	785.3	792.3	839.3	-1.0%	-2.8%	1.5%	0.9%	5.9%
Female	43	Kumamoto	776.7	740.3	747.1	708.7	736.5	810.6	-4.7%	0.9%	-5.1%	3.9%	10.1%
Female	44	Oita	756.6	752.0	748.2	717.0	761.6	792.5	-0.6%	-0.5%	-4.2%	6.2%	4.1%
Female	45	Miyazaki	778.3	784.9	760.0	762.7	780.9	846.5	0.9%	-3.2%	0.4%	2.4%	8.4%
Female	46	Kagoshima	789.7	798.4	788.8	770.7	783.9	844.3	1.1%	-1.2%	-2.3%	1.7%	7.7%
Female	47	Okinawa	742.2	754.1	728.2	701.1	767.0	845.1	1.6%	-3.4%	-3.7%	9.4%	10.2%

*Increasing mortality (plus percent change) was shown in red.

eTable 6. Number of observed deaths, excess deaths, and exiguous deaths* in Japan between 2017 and 2022 (for both sexes)

	2017	2018	2019	2020	2021	2022
All-cause						
Number of deaths (observed)	1340567	1362470	1381093	1372755	1439856	1569050
Excess deaths	2953 to 26535	4607 to 20156	980 to 10860	318 to 8088	13072 to 53667	51056 to 119401
Exiguous deaths	0 to 2357	1266 to 13985	798 to 15026	8840 to 43107	0 to 1795	0 to 0
Malignant neoplasms (C00–C97)						
Number of deaths (observed)	373365	373584	376425	378385	381505	385797
Excess deaths	45 to 1127	5 to 1833	128 to 2521	228 to 2965	411 to 3565	416 to 3625
Exiguous deaths	298 to 3062	237 to 3189	72 to 1627	429 to 2284	0 to 1339	0 to 1184
Diseases of the circulatory system (I00-I99)						
Number of deaths (observed)	351019	352525	350505	345476	357561	381327
Excess deaths	1831 to 15005	1828 to 8706	266 to 3132	316 to 3365	1030 to 13810	6759 to 26188
Exiguous deaths	0 to 178	416 to 5055	417 to 8989	3087 to 15081	3 to 1261	0 to 215
Senility (R54)						
Number of deaths (observed)	101411	109605	121863	132440	152027	179529
Excess deaths	0 to 594	0 to 548	66 to 2138	243 to 2289	2496 to 8139	5294 to 12636
Exiguous deaths	51 to 1669	374 to 3110	37 to 1214	385 to 2288	0 to 119	0 to 238
Suicide (X60-84)						
Number of deaths (observed)	20468	20031	19425	20243	20291	21252
Excess deaths	86 to 938	191 to 1150	170 to 1272	1009 to 2386	216 to 1469	219 to 1345
Exiguous deaths	0 to 585	0 to 265	43 to 328	63 to 794	2 to 555	4 to 556
COVID-19 (U07)						
Number of deaths (observed)	-	-	-	3466	16766	47636

COVID-19, coronavirus disease 2019.

*Excess deaths and exiguous deaths were retrieved from “Excess and Exiguous Deaths Dashboard in Japan” managed by National Institute of Infectious Diseases, Japan (URL: https://exdeaths-japan.org/en/)

(A) COVID-19 case

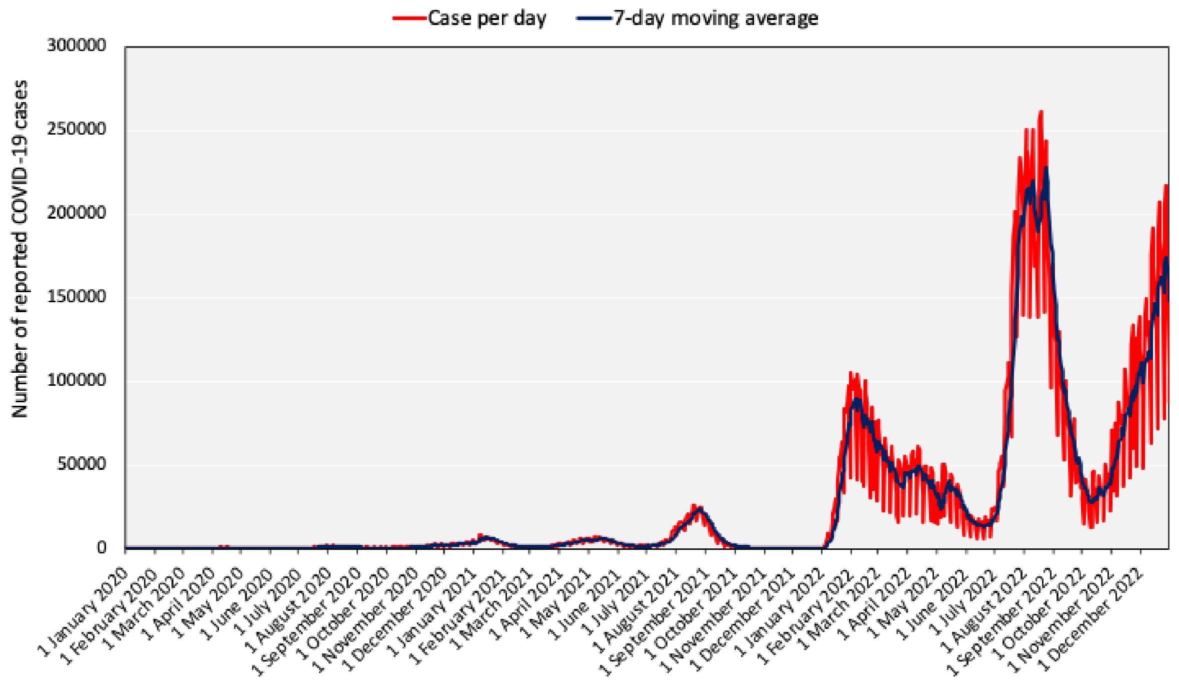

(B) COVID-19 death

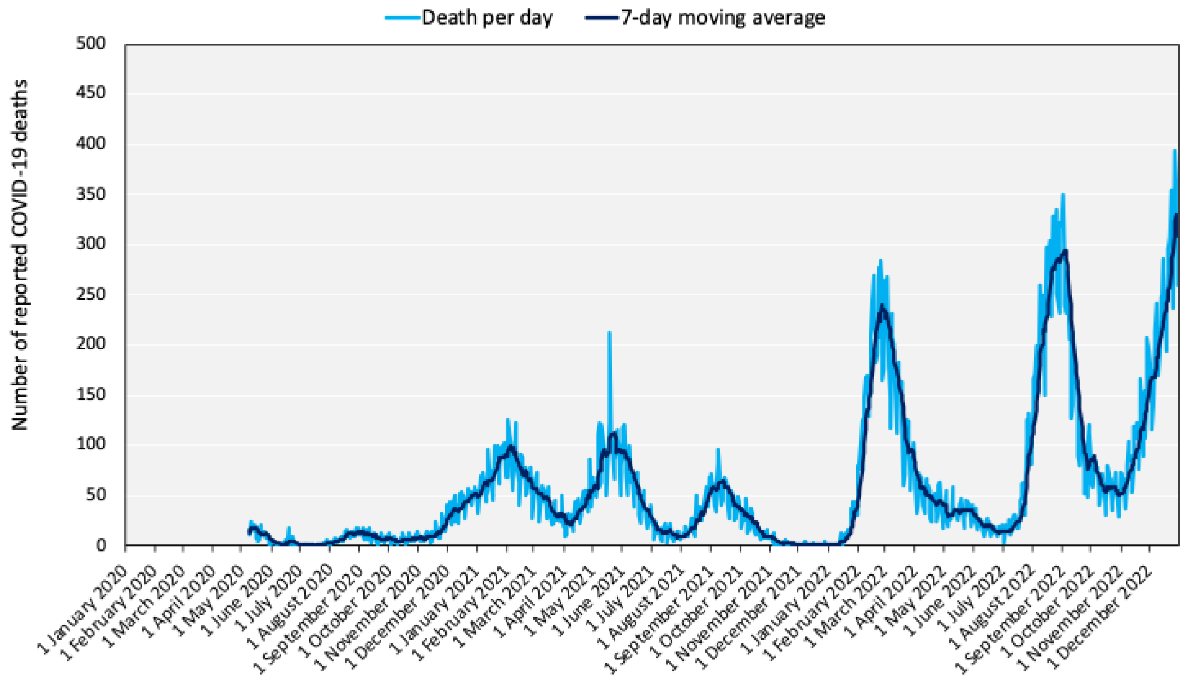

eFigure 1. Trends in the daily number of (**A**) reported COVID-19 cases and (**B**) COVID-19 deaths in Japan between 2020 and 2022

(A) Oral cavity and pharynx (C00-14)

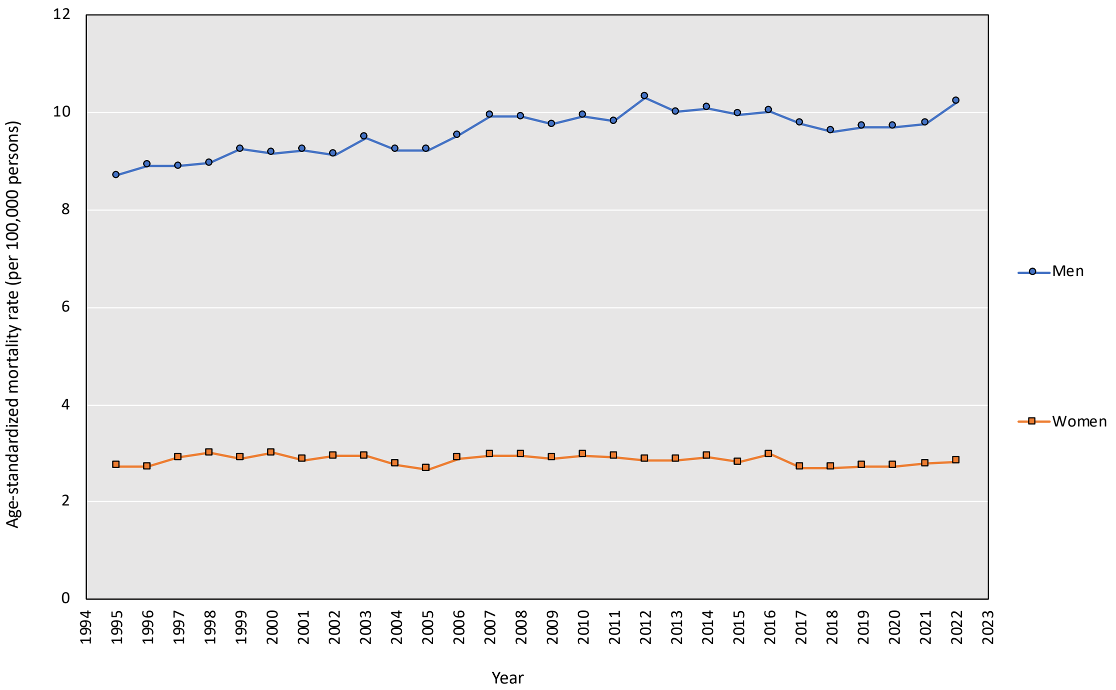

(B) Esophagus (C15)

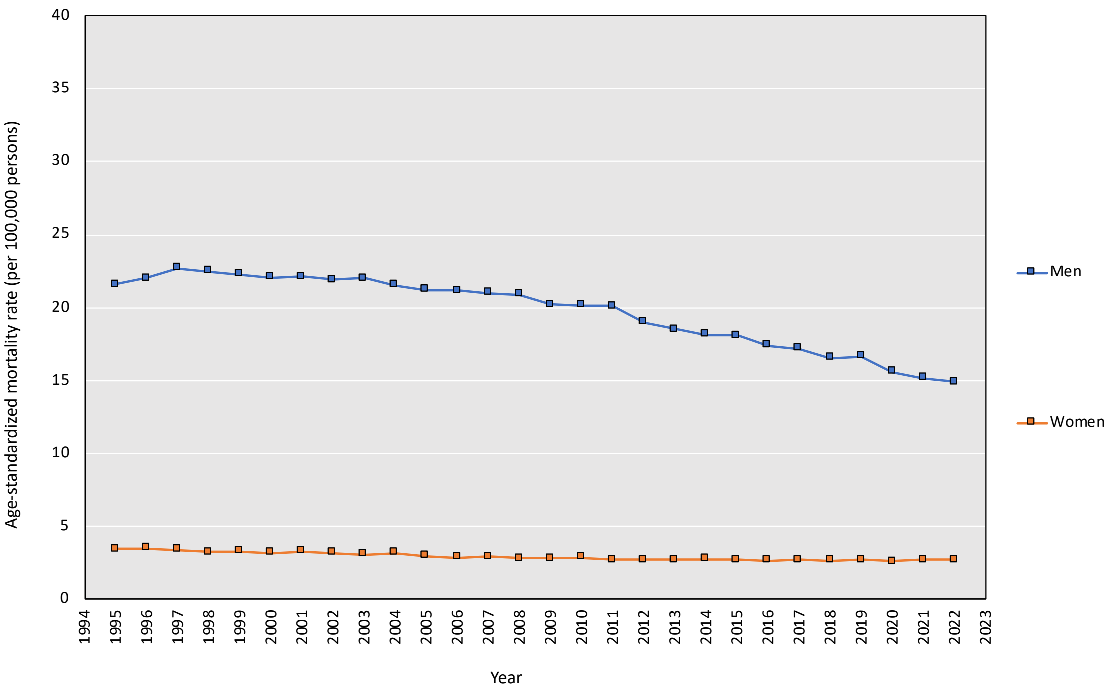

(C) Stomach (C16)

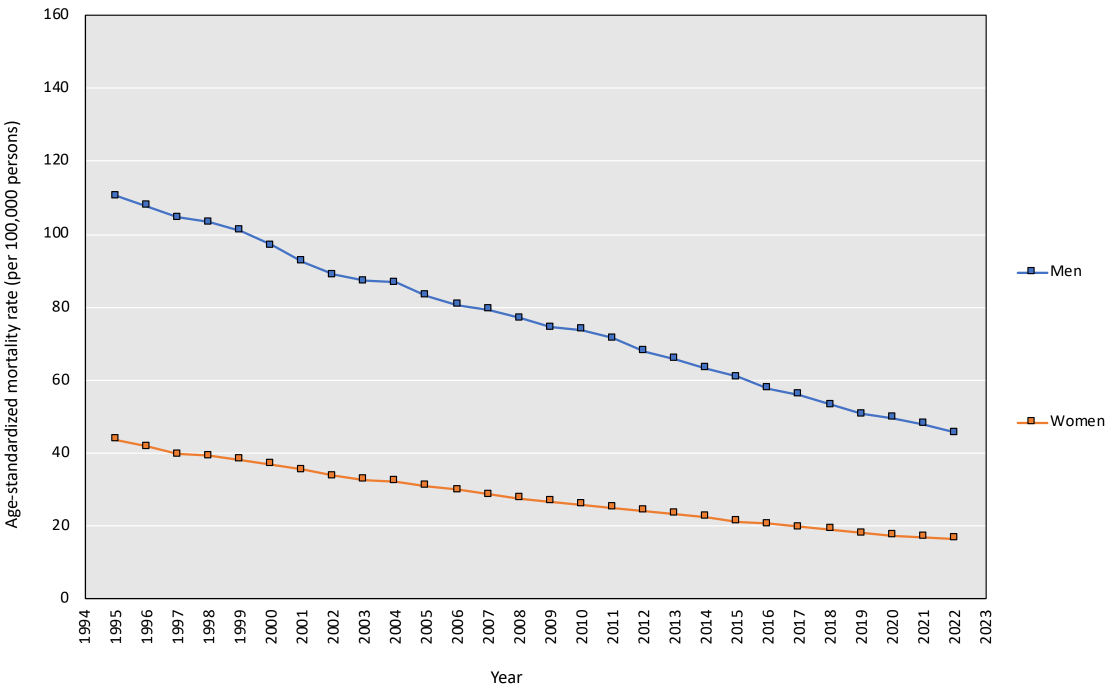

(D) Colon/rectum (C18-20)

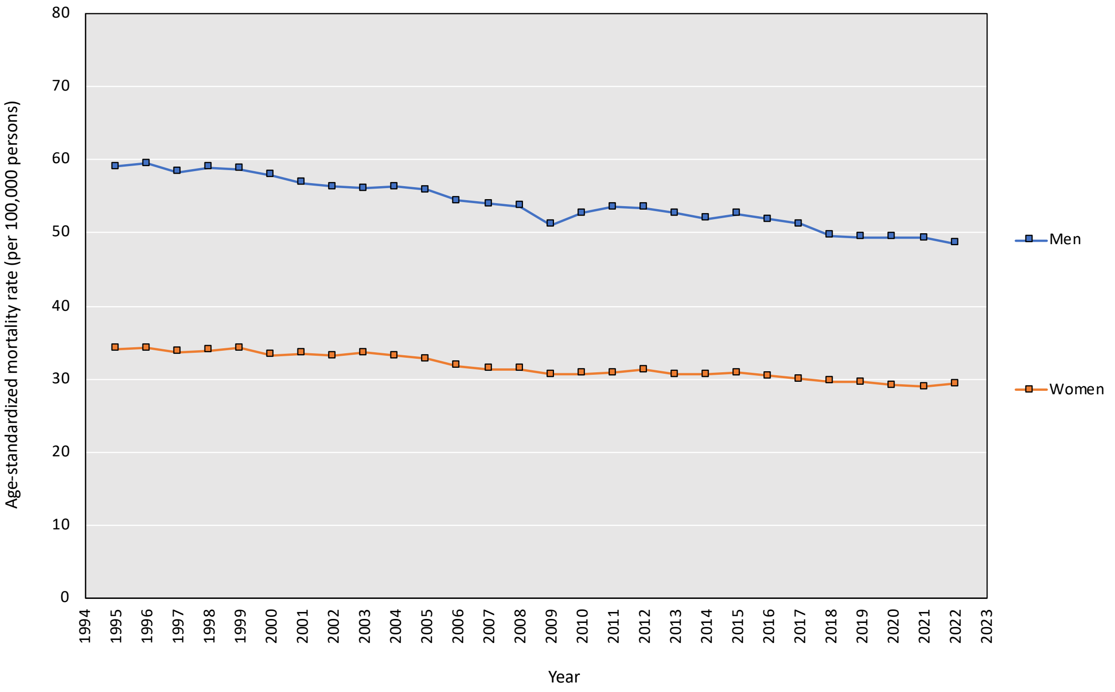

(E) Liver (C22)

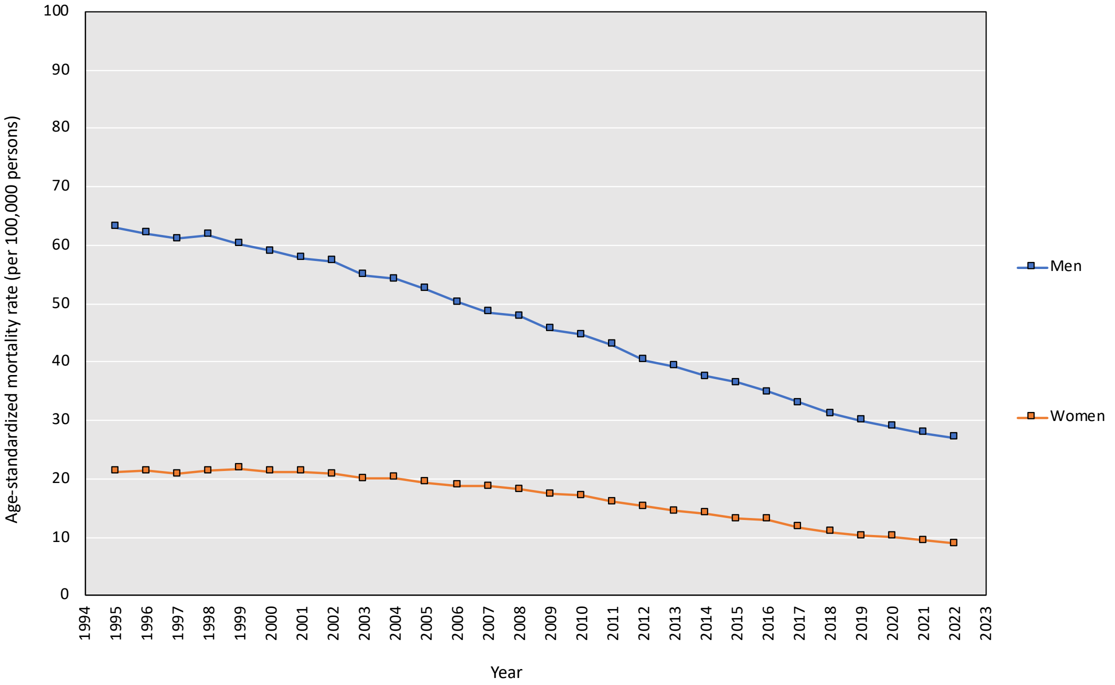

(F) Gallbladder and bile ducts (C23-24)

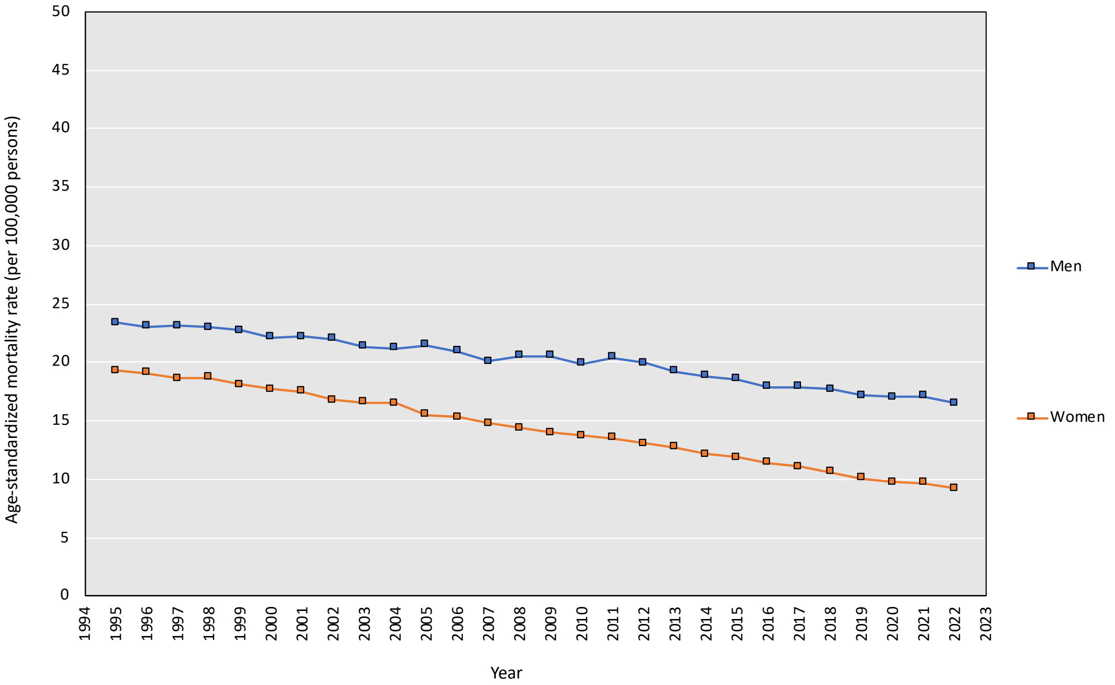

(G) Pancreas (C25)

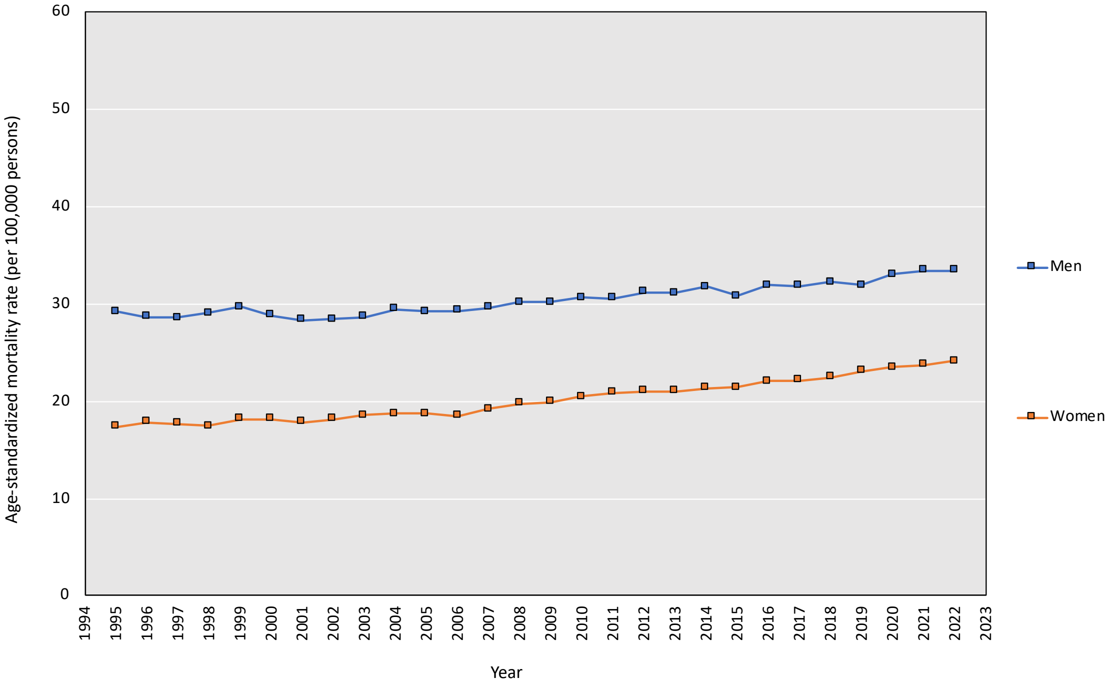

(H) Larynx (C32)

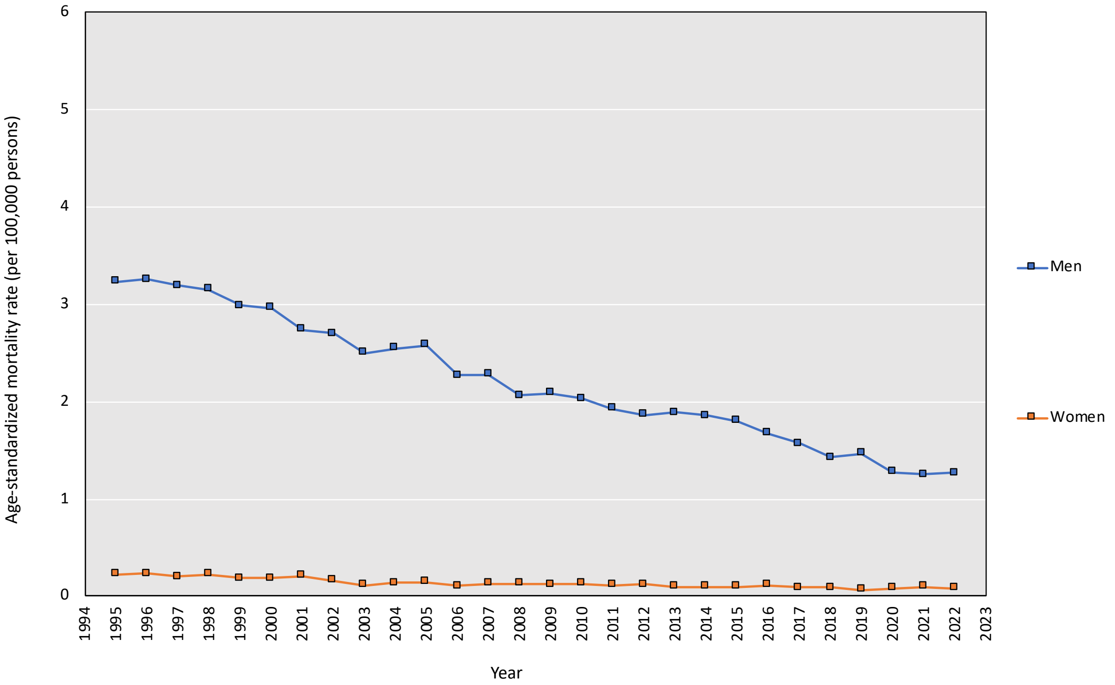

(I) Lung, trachea (C33-34)

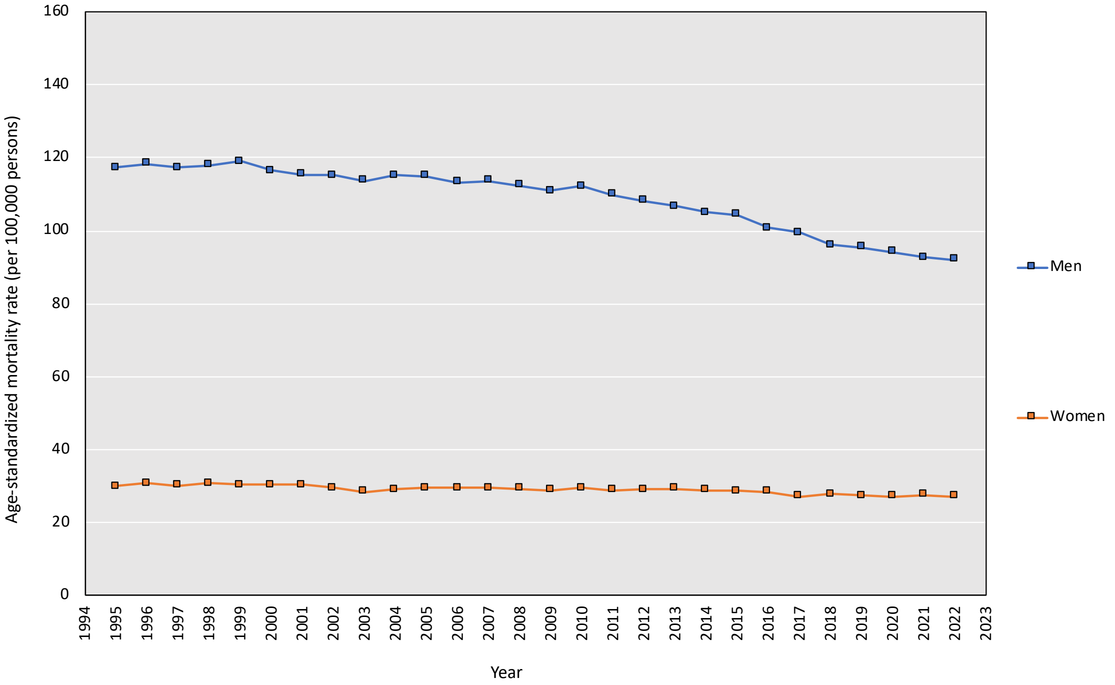

(J) Skin (C43-44)

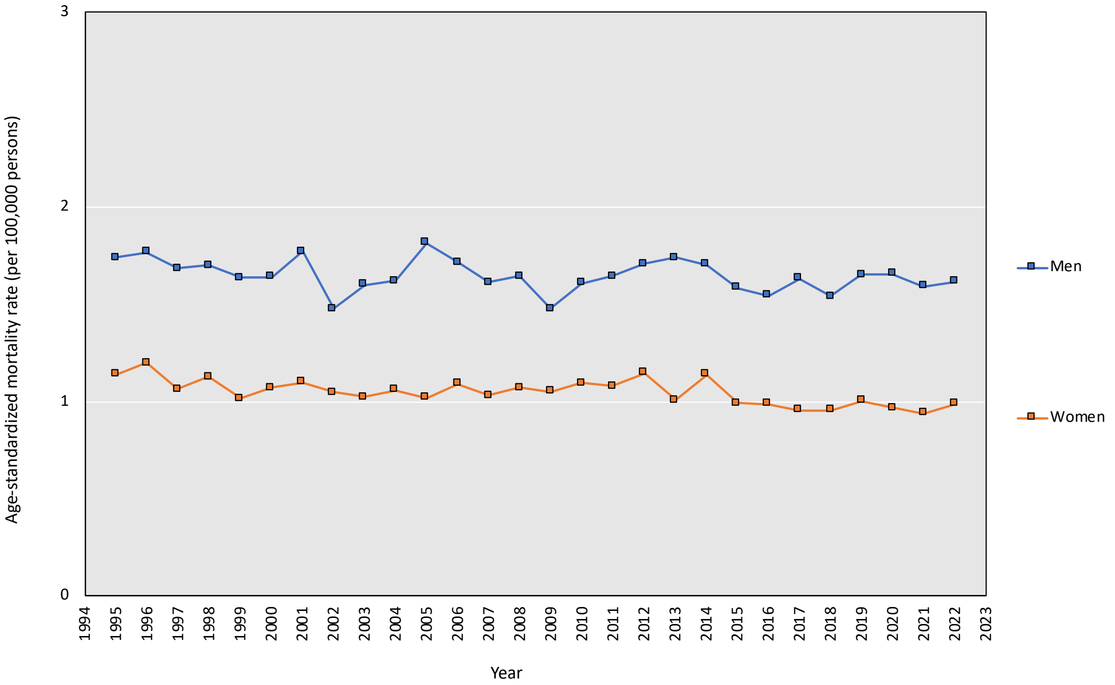

(K) Breast (C50)

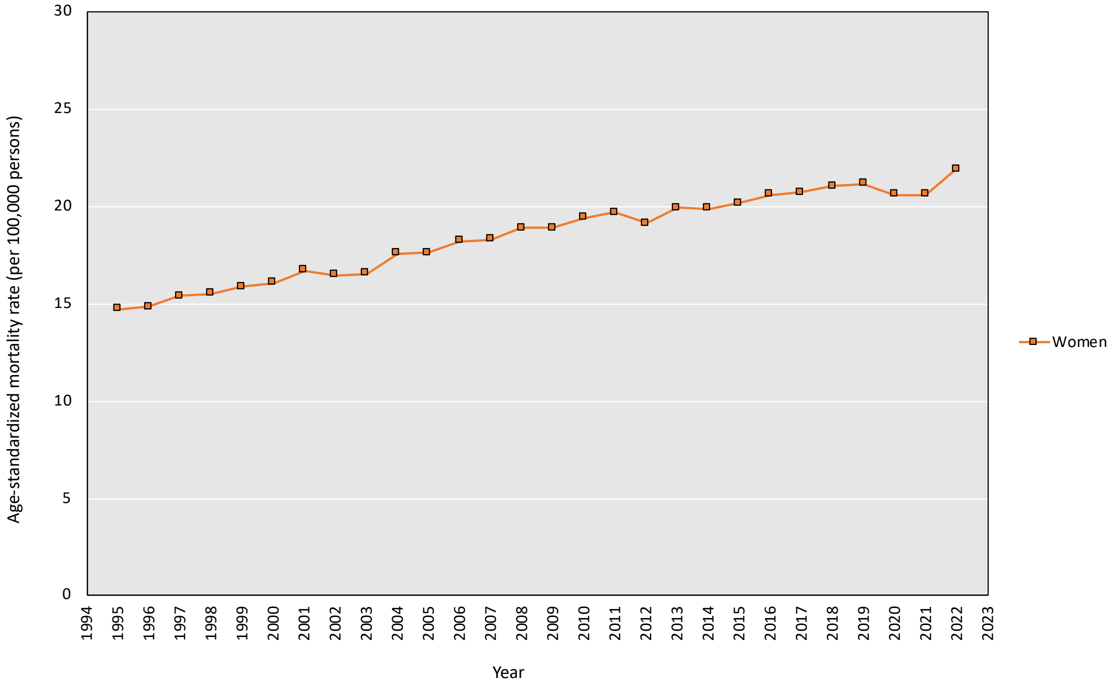

(L) Cervix uteri (C53)

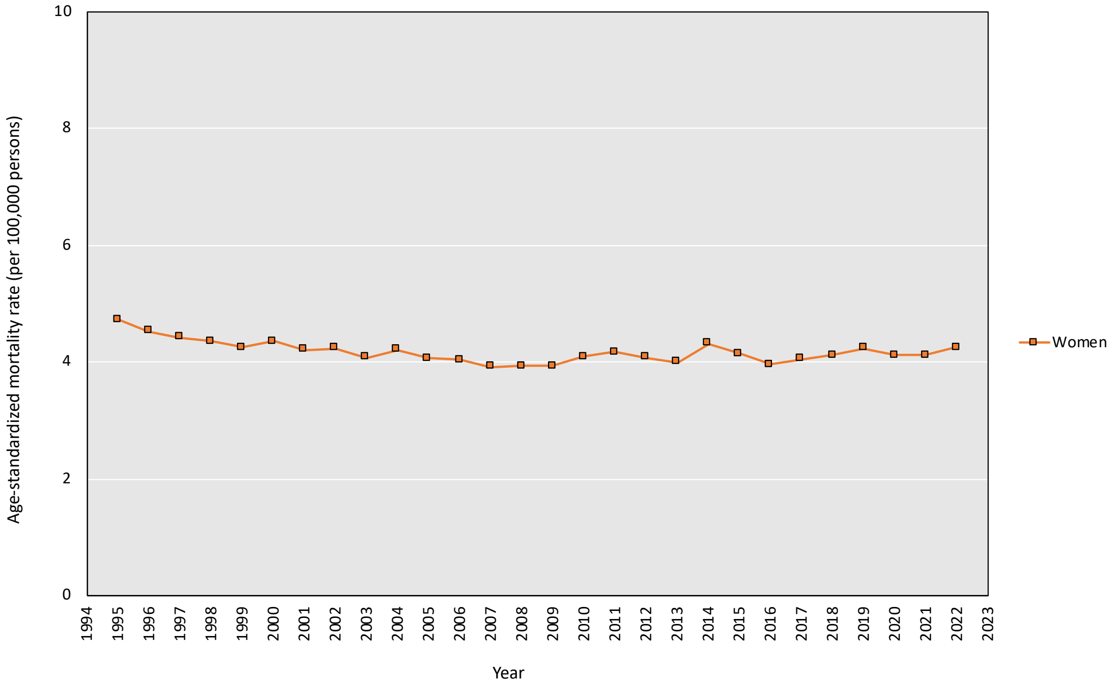

(M) Uterus (C54)

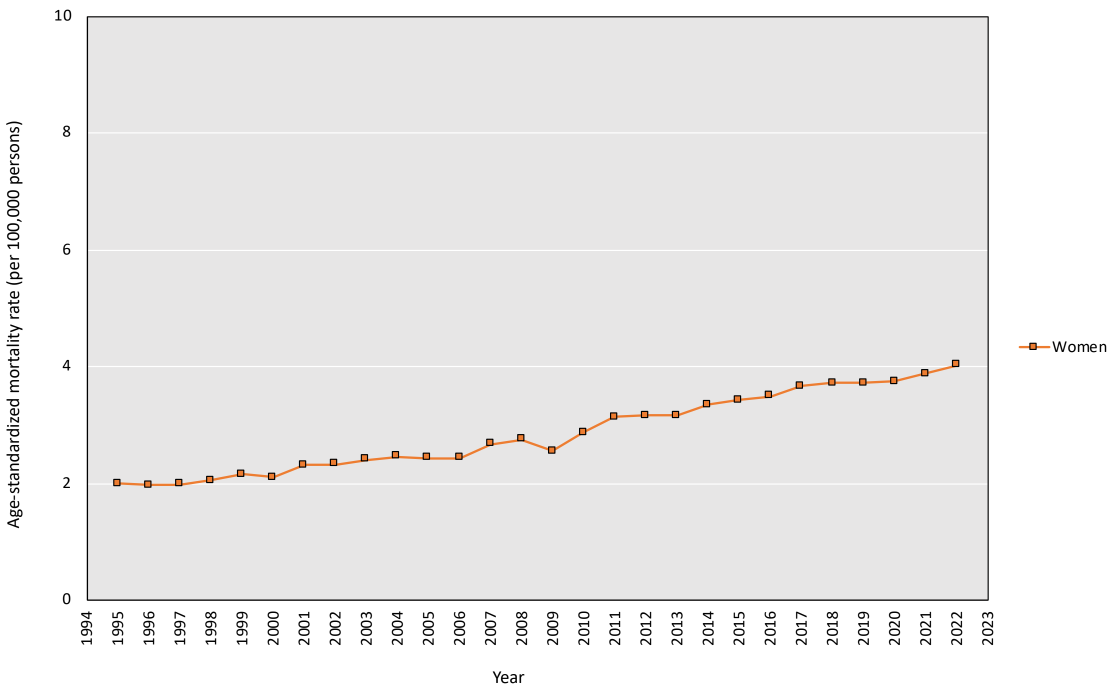

(N) Ovary (C56)

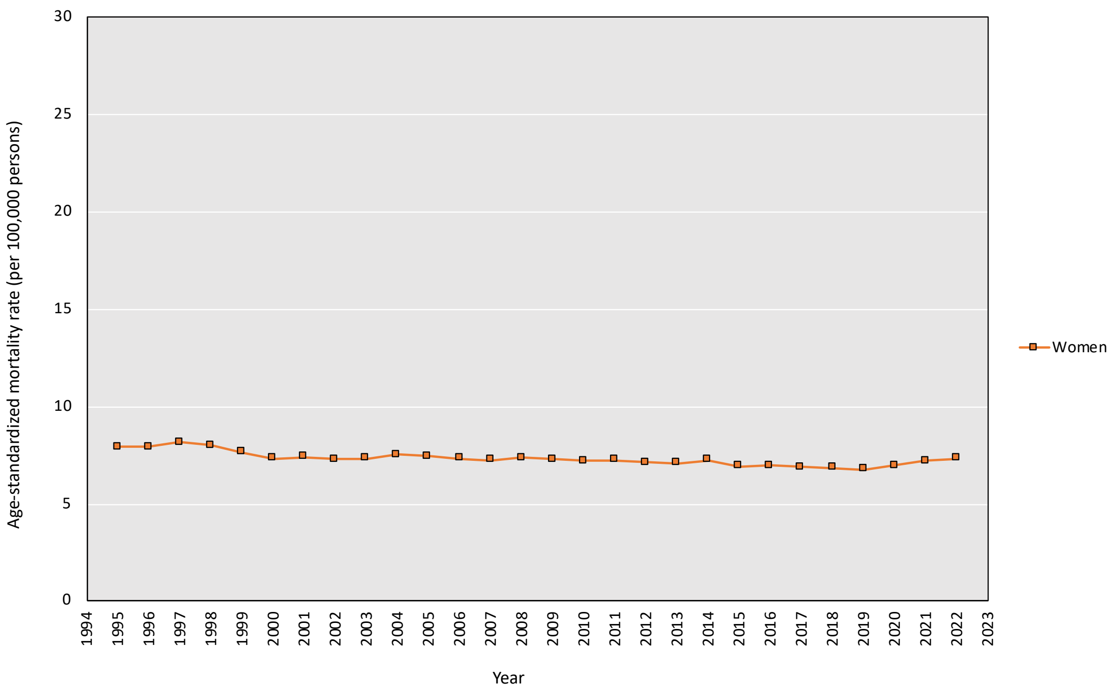

(O) Prostate (C61)

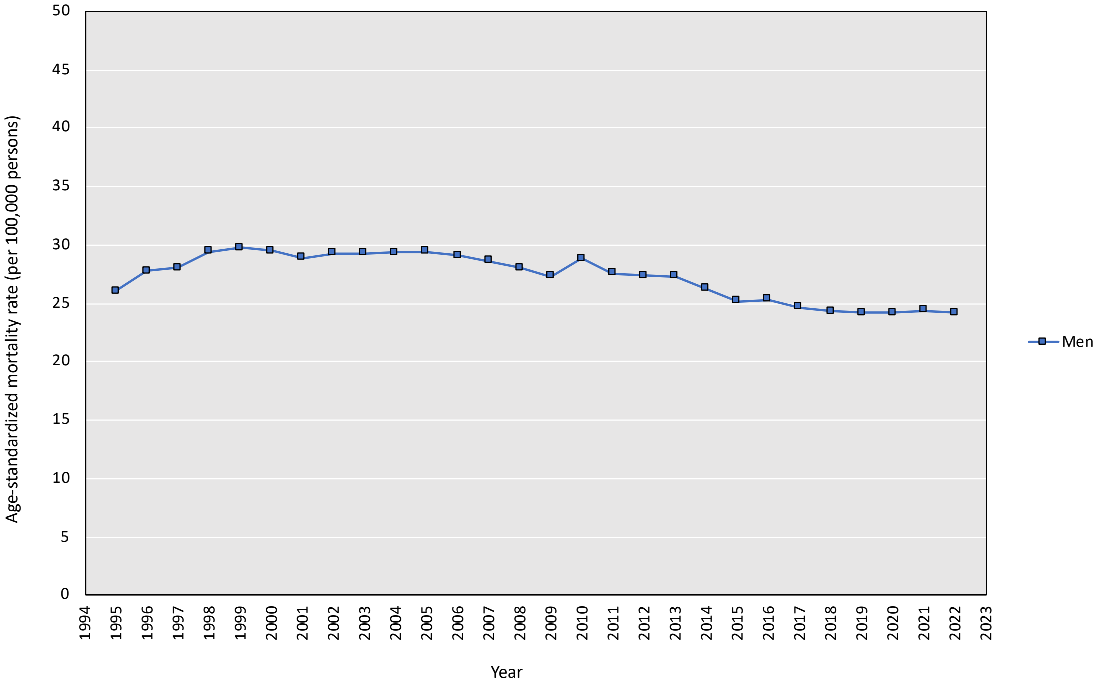

(P) Bladder (C67)

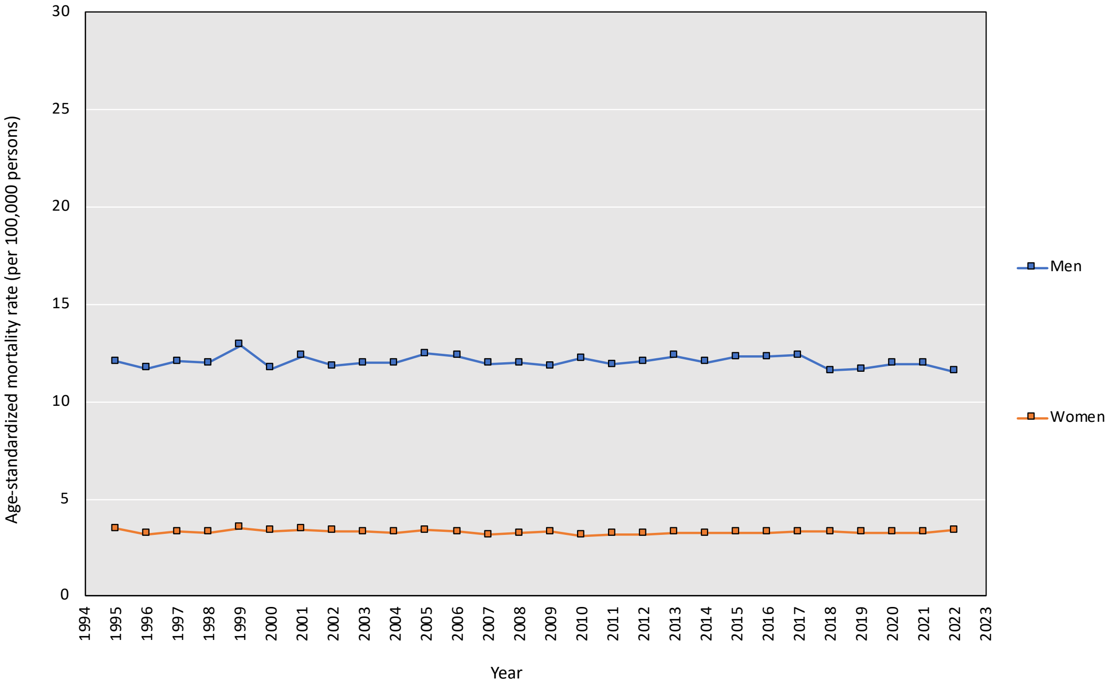

(Q) Brain, nervous system (C70-72)

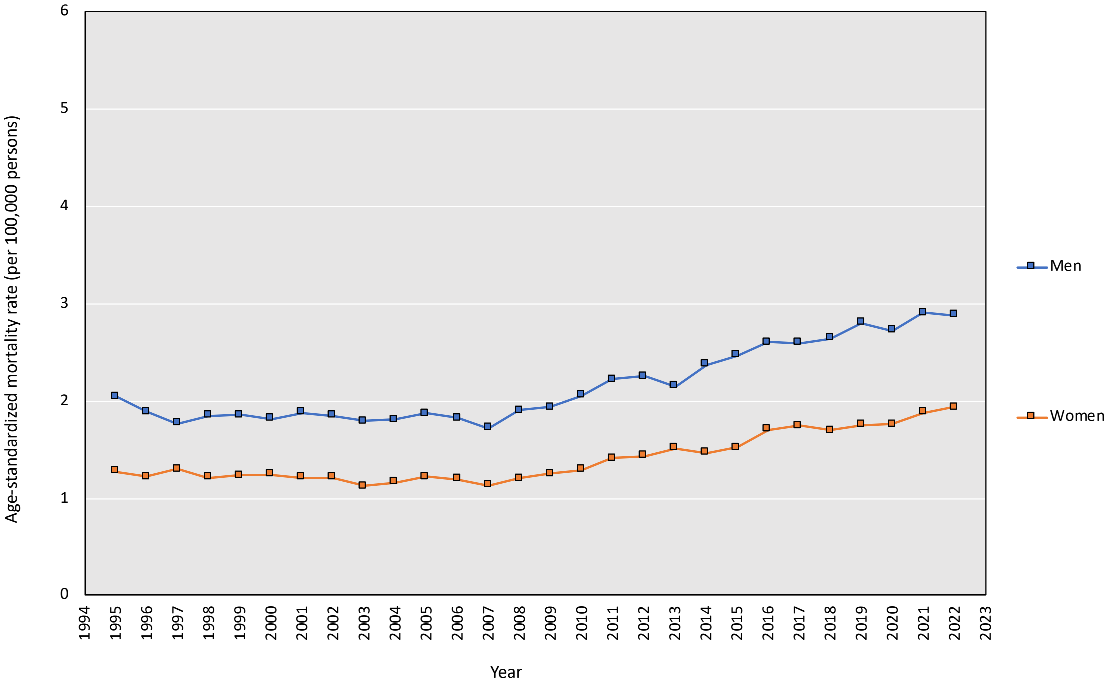

(R) Malignant lymphoma (C81-85, C96)

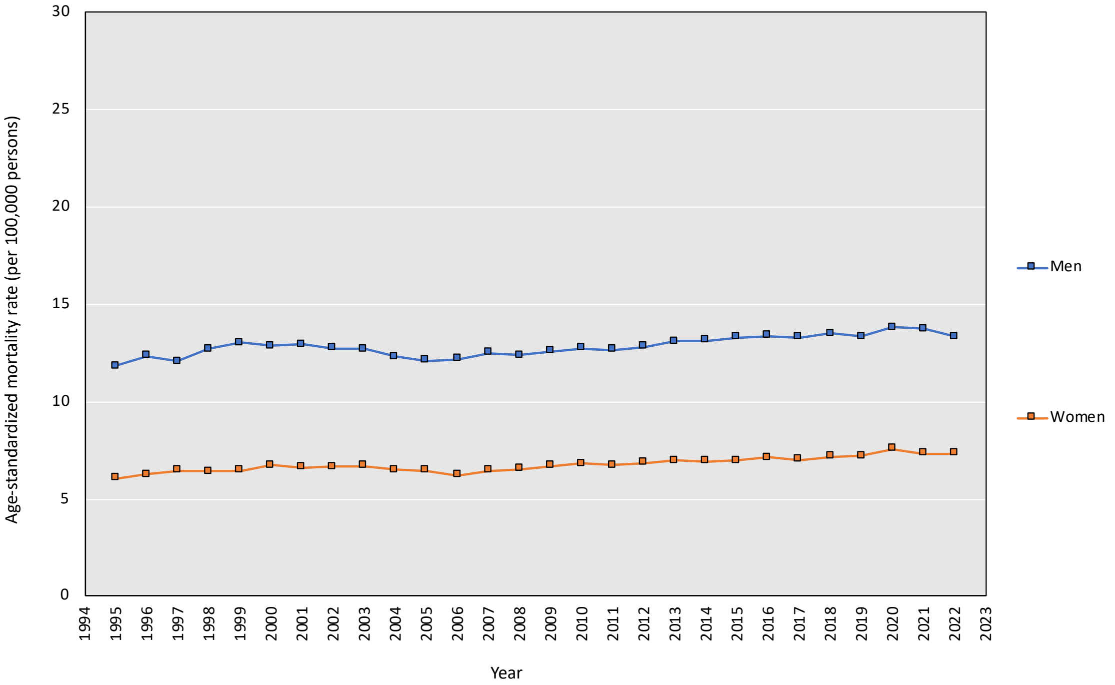

(S) Leukemia (C91-95)

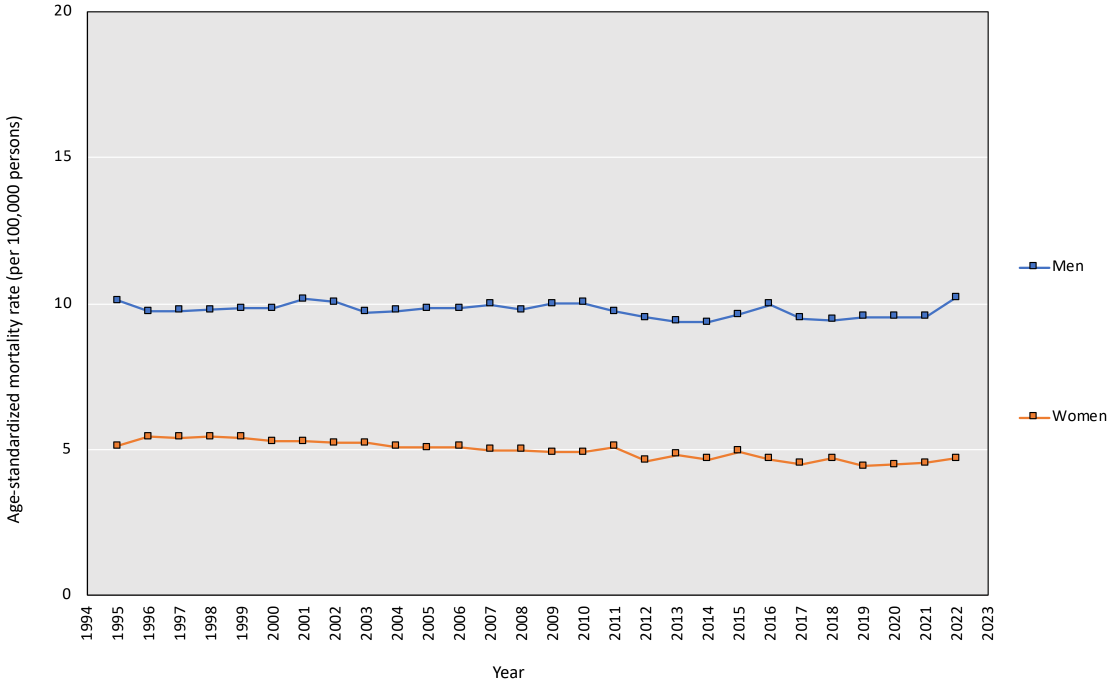

eFigure 2. Trends in cancer age-standardized mortality rates by cancer site between 1995 and 2022
